# Precise Species Identification for *Enterobacter*: a Genome Sequence-Based Study with Reporting of Two Novel Species, *Enterobacter quasiroggenkampii* sp. nov. and *Enterobacter quasimori* sp. nov.

**DOI:** 10.1128/mSystems.00527-20

**Published:** 2020-08-04

**Authors:** Wenjing Wu, Yu Feng, Zhiyong Zong

**Affiliations:** a Center of Infectious Diseases, West China Hospital, Sichuan University, Chengdu, Sichuan, China; b Laboratory of Clinical Microbiology, Department of Laboratory Medicine, West China Second Hospital, Sichuan University, Chengdu, China; c Division of Infectious Diseases, State Key Laboratory of Biotherapy, Chengdu, Sichuan, China; d Center for Pathogen Research, West China Hospital, Sichuan University, Chengdu, Sichuan, China; e Department of Infection Control, West China Hospital, Sichuan University, Chengdu, Sichuan, China; University of Delhi

**Keywords:** *Enterobacter*, *Enterobacter quasiroggenkampii*, *Enterobacter quasimori*, *Enterobacter cloacae*, taxonomy, *Enterobacteriaceae*

## Abstract

The genus *Enterobacter* comprises common pathogens and has a complicated taxonomy. Precise taxonomic assignation lays a foundation for microbiology. In this study, we updated the *Enterobacter* taxonomy based on robust genome analyses. We found that all *Enterobacter* subspecies assignments were incorrect. Enterobacter cloacae subsp. *dissolvens* and Enterobacter hormaechei subsp. *hoffmannii* are species (Enterobacter dissolvens and *Enterobacter hoffmannii*, respectively) rather than subspecies. Enterobacter xiangfangensis, Enterobacter hormaechei subsp. *oharae*, and Enterobacter hormaechei subsp. *steigerwaltii* are not Enterobacter hormaechei subspecies but belong to the same species (Enterobacter xiangfangensis). *Enterobacter timonensis* should be removed to *Pseudenterobacter*, a novel genus. We then reported two novel species, *Enterobacter quasiroggenkampii* and *Enterobacter quasimori*, by genome- and phenotype-based characterization. We also applied the updated taxonomy to curate 1,997 *Enterobacter* genomes in GenBank. Species identification was changed following our updated taxonomy for the majority of publicly available strains (1,542, 77.2%). The most common *Enterobacter* species was *E. xiangfangensis*. We identified 14 novel tentative *Enterobacter* genomospecies. This study highlights that updated and curated taxonomic assignments are the premise of correct identification.

**IMPORTANCE** *Enterobacter* species are major human pathogens. Precise species identification lays a foundation for microbiology, but the taxonomy of *Enterobacter* is complicated and confusing. In this study, first, we significantly updated the taxonomy of *Enterobacter* by rigorous genome analyses and found that all subspecies assignments of *Enterobacter* were incorrect. Second, we characterized and reported two novel *Enterobacter* species with clinical significance. Third, we curated 1,997 *Enterobacter* genome sequences deposited in GenBank and found that the species identification of most *Enterobacter* strains needed to be corrected. Fourth, we found that the most common *Enterobacter* species seen in clinical samples is Enterobacter xiangfangensis rather than Enterobacter cloacae. Fifth, we identified 14 tentative novel *Enterobacter* and 18 tentative novel non-*Enterobacter* species. This study highlights that updated and curated taxonomic assignments are the premise of correct species identification. We recommend that future *Enterobacter* studies need to use the updated taxonomy to avoid misleading information.

## Abstract (precis)

*Enterobacter* species are major human pathogens. Precise species identification lays a foundation for microbiology, but the taxonomy of *Enterobacter* is complicated and confusing. In this study, first, we significantly updated the taxonomy of *Enterobacter* by rigorous genome analyses and found that all subspecies assignments of *Enterobacter* were incorrect. Second, we characterized and reported two novel *Enterobacter* species with clinical significance. Third, we curated 1,997 *Enterobacter* genome sequences deposited in GenBank and found that the species identification of most *Enterobacter* strains needed to be corrected. Fourth, we found that the most common *Enterobacter* species seen in clinical samples is Enterobacter xiangfangensis rather than Enterobacter cloacae. Fifth, we identified 14 tentative novel *Enterobacter* and 18 tentative novel non-*Enterobacter* species. This study highlights that updated and curated taxonomic assignments are the premise of correct species identification. We recommend that future *Enterobacter* studies need to use the updated taxonomy to avoid misleading information.

## INTRODUCTION

*Enterobacter* is a genus of Gram-negative, non-spore-forming bacteria of the family *Enterobacteriaceae* and is close to the genera *Leclercia* and *Lelliottia* (1). *Enterobacter* is widely distributed in nature and is a well-known pathogen for plant diseases (2). In addition, *Enterobacter* is also part of the commensal microflora of the human gut (3, 4). A few *Enterobacter* species, e.g., Enterobacter cloacae, Enterobacter asburiae, and Enterobacter hormaechei, are common pathogens of human infections, particularly hospital-acquired infections (5). Precise species and subspecies assignation of bacterial isolates lays a foundation for understanding the epidemiology, pathogenesis, and microbiological features of bacteria and has important implications for diagnosis, treatment, prognosis, and prevention. Clinical microbiology laboratories commonly use phenotype-based tests including automated microbiology systems such as Vitek II and matrix-assisted laser desorption ionization–time of flight mass spectrum (MALDI-TOF) for species identification, which usually identify *Enterobacter* clinical isolates as E. cloacae or sometimes as *E. asburiae*, *E. hormaechei*, or Enterobacter kobei. However, it is known that phenotype-based tests cause misidentification of *Enterobacter* and are unreliable for precise species identification (3). DNA-DNA hybridization (DDH) with a ≥70% cutoff has long been used as the “gold standard” for species delineation (6), but DDH is error-prone and has low reproducibility. 16S rRNA gene sequence identity has therefore been used as a proxy of DDH. However, it is well known that analysis on 16S rRNA gene sequence is insufficient for accurate bacterial species assignation (7). As the cost has been massively reduced, whole-genome sequencing has been increasingly used in clinical microbiology laboratories, which allows precise species identification (8). The pairwise average nucleotide identity (ANI) with a ≥96% cutoff and *in silico* DNA-DNA hybridization (isDDH, also called digital DDH [dDDH]) with a ≥70.0% cutoff mimic traditional DDH and have been widely used for precise species identification (9–11).

Updated and curated taxonomic assignment is the premise of precise species identification, but the taxonomy of *Enterobacter* is complicated by the fact that many species that used to belong to *Enterobacter* have been moved out to other genera. For instance, Enterobacter aerogenes, Enterobacter agglomerans, and Enterobacter cowanii have been moved to the genus *Klebsiella*, *Pantoea*, and *Kosakonia*, respectively (4, 12, 13). Until now, the *Enterobacter* genus has been comprised of 19 species plus 6 subspecies with validly published names (Table 1) (14). Genome sequences of type strains of all *Enterobacter* species and subspecies except Enterobacter cloacae subsp. *dissolvens* are available. However, previous studies have found that the species and subspecies assignment within the genus *Enterobacter* is problematic (15). In particular, subspecies assignments within *Enterobacter* have been defined based on low-resolution analytical methods (16, 17) and may need to be carefully examined (18). For instance, Enterobacter xiangfangensis has been validly published as a species (19) but a recent study has proposed it as a subspecies of *E. hormaechei* (Enterobacter hormaechei subsp. *xiangfangensis*) based on an *in silico* analysis (18). This causes confusion, and each taxon should bear only one correct assignation (20).

**TABLE 1:** Classification and nomenclature of the genus *Enterobacter* as of April 2020

Species	Type strain	Accession no. or current species name
Species (*n* = 19, including 6 subspecies)		
*Enterobacter asburiae*^*a*^	JCM 6051	CP011863
*Enterobacter bugandensis*	EB-247	FYBI00000000
*Enterobacter cancerogenus*	ATCC 35316	ERR1854846
*Enterobacter chengduensis*	WCHECl-C4	MTSO00000000
*Enterobacter chuandaensis*	090028	QZCS00000000
*Enterobacter cloacae*	ATCC 13047	CP001918
*E. cloacae* subsp. *cloacae*	ATCC 13047	CP001918
*E. cloacae* subsp. *dissolvens*	ATCC 23373	WJWQ00000000^*d*^
*Enterobacter hormaechei*	ATCC 49162	MKEQ00000000
*E. hormaechei* subsp. *hormaechei*	ATCC 49162	MKEQ00000000
*E. hormaechei* subsp. *hoffmannii*	DSM 14563	CP017186
*E. hormaechei* subsp. *oharae*	DSM 16687	CP017180
*E. hormaechei* subsp. *steigerwaltii*	DSM 16691	CP017179
*Enterobacter huaxiensis*	090008	QZCT00000000
*Enterobacter kobei*	ATCC BAA-260	CP017181
*Enterobacter ludwigii*	EN-119	CP017279
*Enterobacter mori*^*b*^	LMG 25706	GL890773
*Enterobacter oligotrophica*	CCA6	AP019007
*Enterobacter quasihormaechei*	WCHEs120003	SJON00000000
*Enterobacter roggenkampii*	DSM 16690	CP017184
*Enterobacter sichuanensis*	WCHECl1597	POVL00000000
*Enterobacter soli*	ATCC BAA-2102	LXES00000000
*Enterobacter timonensis*	mt20	FCOP00000000
*Enterobacter wuhouensis*	WCHEs120002	SJOO00000000
*Enterobacter xiangfangensis*^*c*^	LMG 27195	CP017183
* *		
Species rejected (*n* = 4)		
*Enterobacter muelleri*^*a*^	JM-458	*Enterobacter asburiae*
*Enterobacter siamensis*^*e*^	C2361	
*Enterobacter tabaci*^*b*^	YIM Hb-3	*Enterobacter mori*
*Enterobacter taylorae*^*f*^	ATCC 35317	*Enterobacter cancerogenus*
* *		
Species listed in LPSN but moved out of *Enterobacter* (*n* = 19)		
*Enterobacter aerogenes*	ATCC 13048	*Klebsiella aerogenes*
*Enterobacter agglomerans*	ATCC 27155	*Pantoea agglomerans*
*Enterobacter amnigenus*	ATCC 33072	*Lelliottia amnigena*
*Enterobacter arachidis*	KCTC 22375	*Kosakonia arachidis*
*Enterobacter cowanii*	CCUG 45998	*Kosakonia cowanii*
*Enterobacter gergoviae*	ATCC 33028	*Pluralibacter gergoviae*
*Enterobacter helveticus*	JCM 16470	*Cronobacter helveticus*
*Enterobacter intermedius*	ATCC 33110	*Kluyvera intermedia*
*Enterobacter massiliensis*	JC163	*Metakosakonia* *massiliensis*
*Enterobacter nimipressuralis*	CIP 104980	*Lelliottia nimipressuralis*
*Enterobacter oryzae*	LMG 24251	*Kosakonia oryzae*
*Enterobacter oryzendophyticus*	LMG 26432	*Kosakonia oryzendophytica*
*Enterobacter oryziphilus*	LMG 26429	*Kosakonia oryziphila*
*Enterobacter pulveris*	DSM 19144	*Cronobacter pulveris*
*Enterobacter pyrinus*	ATCC 49851	*Pluralibacter pyrinus*
*Enterobacter* *radicincitans*	CIP 108468	*Kosakonia radicincitans*
*Enterobacter sacchari*	CGMCC 1.12102	*Kosakonia sacchari*
*Enterobacter sakazakii*	ATCC 29544	*Cronobacter sakazakii*
*Enterobacter turicensis*	DSM 18397	*Cronobacter zurichensis*

a Enterobacter muelleri is a later synonym of Enterobacter asburiae (42).

b Enterobacter tabaci (type strain YIM Hb-3) is a later synonym of Enterobacter mori (15).

c The species status of Enterobacter xiangfangensis has been proposed as a subspecies of Enterobacter hormaechei rather than a valid species (18). However, its type strain has only 94.48% ANI and 60.0% isDDH with *E. hormaechei* type strain ATCC 49162^T^ (GenBank accession no. MKEQ00000000). Therefore, it is clear that *E. xiangfangensis* and *E. hormaechei* are two different species.

d The genome sequencing was performed in the present study.

e Enterobacter siamensis is rejected as the 16S rRNA sequence of its type strain available in collections does not match its record in GenBank (43).

f Enterobacter taylorae is a later synonym of Enterobacter cancerogenus (44).

In this study, we performed whole-genome sequencing for the type strain and report here that E. cloacae subsp. *dissolvens* is actually an independent species rather than a subspecies of E. cloacae. We then performed genome-based comparison and a phylogenetic analysis to clarify the exact taxonomic positions of the subspecies of *E. hormaechei*. We found that *E. hormaechei* and *E. xiangfangensis* are indeed different species, while *E. hormaechei* subsp. *steigerwaltii* and *E. hormaechei* subsp. *oharae* are later synonyms of *E. xiangfangensis*. In addition, *E. hormaechei* subsp. *hoffmannii* is a species rather than a subspecies. We also found that Enterobacter timonensis should be removed to a novel genus with the proposed name *Pseudenterobacter*. We also identified and characterized two novel *Enterobacter* species, which were distinct from all hitherto-known species, by both genome- and phenotype-based methods. We then used the updated taxonomy of *Enterobacter* to review and curate the species assignment of all *Enterobacter* genomes (*n* = 1,997) in GenBank to correct the corresponding misleading information. We found that the majority of *Enterobacter* strains with whole-genome sequences available are not E. cloacae but *E. xiangfangensis*. We also found that there are 14 tentative novel *Enterobacter* species based on genome analysis, which need to be further studied using phenotype-based methods to establish their species status.

## RESULTS

### E. cloacae subsp. *dissolvens* is a species rather than a subspecies and should be renamed Enterobacter dissolvens.

Whole-genome sequencing for strain ATCC 23373^T^ generated 2,908,248 reads and 0.87 gigabases, which were assembled into a 4.84-Mb draft genome containing 51 contigs ≥200 bp in length (*N*_50_, 415,836 bp) with a 55.16% GC content. No contamination was identified in the genomes. The *gyrB*, *rpoB*, *infB*, and *atpD* sequences were identical to those of strain ATCC 23373^T^ previously deposited in GenBank (accession no. JX424979, JX425238, JX425108, and JX424849, respectively), suggesting that this strain was indeed strain ATCC 23373^T^. The ANI value between strain ATCC 23373^T^ and E. cloacae subsp. *cloacae* ATCC 13047^T^ (GenBank accession no. CP001918) was 94.79% (ATCC 13047^T^ versus ATCC 23373^T^) or 94.92% (vice versa), below the 96% ANI cutoff to define a bacterial species (9). The isDDH value between the type strains was 62.0%, lower than the 70.0% cutoff to define a bacterial species (10). Both ANI and isDDH analyses indicate that E. cloacae subsp. *dissolvens* should be considered a species different from E. cloacae subsp. *cloacae*. In addition, the ANI and isDDH values between strain ATCC 23373^T^ and type strains of all other *Enterobacter* species are <95% and <70%, respectively (see Table S1 in the supplemental material). We therefore proposed that E. cloacae subsp. *dissolvens* should be elevated to the species level as Enterobacter dissolvens sp. nov. (type strain ATCC 23373^T^ = CIP 105586^T^ = JCM 6049^T^ = LMG 2683^T^).

10.1128/mSystems.00527-20.4 TABLE S1 Average nucleotide identity (ANI)/*in silico* DNA-DNA hybridization (isDDH) values between type strains of species belonging to the genus *Enterobacte*r, *Lelliottia*, and *Leclercia*. Download Table S1, DOCX file, 0.02 MB. Copyright © 2020 Wu et al. 2020 Wu et al. This content is distributed under the terms of the Creative Commons Attribution 4.0 International license.

### Enterobacter xiangfangensis is not a subspecies of *E. hormaechei*.

The core gene-based phylogenomic tree (see Fig. S1 in the supplemental material) demonstrated that the type strains of *E. xiangfangensis* and other *E. hormaechei* subspecies formed a clade, which was distinct from all other *Enterobacter* species. This suggests that *E. xiangfangensis* and the *E. hormaechei* subspecies are indeed closely related. Within this *E. hormaechei* clade, *E. hormaechei* subsp. *oharae*, *E. hormaechei* subsp. *steigerwaltii*, and *E. xiangfangensis* were clustered together, while the other two subspecies each appeared to form a distinct branch. The ANI values between the strain *E. hormaechei* subsp. *hormaechei* ATCC 49162^T^, which is also the type strain of the species *E. hormaechei*, and the type strains of other subspecies and *E. xiangfangensis* range from 94.13% to 94.79% (Table 2), which are below the 96% ANI cutoff to define a bacterial species (9). The isDDH value between *E. hormaechei* subsp. *hormaechei* ATCC 49162^T^ and the type strains of other subspecies and *E. xiangfangensis* ranges from 58.0% to 62.5% (Table 2), also lower than the 70% cutoff to define a bacterial species. Both ANI and isDDH analyses clearly indicate that three other subspecies (*E. hormaechei* subsp. *steigerwaltii*, *E. hormaechei* subsp. *oharae*, and *E. hormaechei* subsp*. hoffmannii*) and *E. xiangfangensis* actually do not belong to *E. hormaechei* and should not be considered subspecies of *E. hormaechei*.

**TABLE 2:** The ANI and isDDH values between type strains of Enterobacter hormaechei “subspecies”^*a*^

“Subspecies”	ANI/isDDH, %, for “subspecies”:				
	*hormaechei*	*hoffmannii*	*oharae*	*steigerwaltii*	*xiangfangensis*
*hormaechei*		94.09/58.0	94.79/62.5	94.71/61.7	94.47/60.0
*hoffmannii*	94.13/58.0		95.59/66.9	95.60/66.5	95.71/66.6
*oharae*	94.79/62.5	95.69/66.9		**97.38/80.8**	**97.01/76.2**
*steigerwaltii*	94.56/61.7	95.39/66.5	**97.16/80.8**		**96.62/75.8**
*xiangfangensis*	94.48/60.0	95.61/66.6	**96.88/76.2**	**96.84/75.8**	

a “Subspecies” and strains: *E. hormaechei* subsp. hormaechei ATCC 49162^T^; *E. hormaechei* subsp. *hoffmannii* DSM 14563^T^; *E. hormaechei* subsp. *oharae* DSM 16687^T^; *E. hormaechei* subsp. *steigerwaltii* DSM 16691^T^; *E. xiangfangensis* LMG 27195^T^. Pairwise ANI and isDDH values above the cutoff to define a bacterial species are highlighted in bold.

10.1128/mSystems.00527-20.2 FIG S1 A phylogenetic tree based on the concatenated nucleotide sequence of core genes of type strains of *Enterobacter* species and subspecies (listed in Table 1) as of April 2020 before the study. Strains and their nucleotide accession numbers are listed alongside the names of species. The tree was inferred using the maximum likelihood method under the GTRGAMMA model with a 1,000-bootstrap test, and branches with support over 50% are indicated by gradients. Bar, value indicates the nucleotide substitutions per site. Download FIG S1, TIF file, 2.7 MB. Copyright © 2020 Wu et al. 2020 Wu et al. This content is distributed under the terms of the Creative Commons Attribution 4.0 International license.

### Enterobacter hormaechei subsp. *oharae* and Enterobacter hormaechei subsp. *steigerwaltii* are not subspecies of Enterobacter hormaechei but are later synonyms of Enterobacter xiangfangensis.

Pairwise ANI values among type strains of *E. hormaechei* subsp. *oharae* (strain DSM 16687^T^), *E. hormaechei* subsp. *steigerwaltii* (DSM 16691^T^), and *E. xiangfangensis* (LMG 27195^T^) were all ≥96.62%, and the pairwise isDDH values of the three strains were all ≥75.8% (Table 2). Both the ANI and isDDH values among the three strains were well above the cutoffs to define a bacterial species, indicating that the three type strains belong to a common species. The fact that ANI and isDDH values among *E. xiangfangensis*, *E. hormaechei* subsp. *oharae*, and *E. hormaechei* subsp. *steigerwaltii* are above the cutoff to define bacterial species has also been noticed before (18) and is used as the evidence that *E. xiangfangensis* is a subspecies of *E. hormaechei* (18). As demonstrated above, *E. hormaechei* subsp. *oharae* and *E. hormaechei* subsp. *steigerwaltii* do not belong to *E. hormaechei* in fact. Therefore, the >96% ANI and >70% isDDH values between *E. xiangfangensis* and *E. hormaechei* subsp. *oharae* or *E. hormaechei* subsp. *steigerwaltii* cannot be used as the evidence to reject the species status of *E. xiangfangensis* but provide the proof that the three “subspecies” actually belong to a common species.

### Enterobacter hormaechei subsp. *hoffmannii* is not a subspecies of Enterobacter hormaechei but is a novel species.

The ANI values between the type strain of *E. hormaechei* subsp. *hoffmannii* (DSM 14563^T^) and type strains of *E. hormaechei* subsp. *oharae*, *E. hormaechei* subsp. *steigerwaltii*, and *E. xiangfangensis* range from 95.59% to 95.71% (Table 2), which fall into the 95 to 96% inconclusive zone of defining a bacterial species (9, 21). The isDDH value between *E. hormaechei* subsp. *hoffmannii* strain DSM 14563^T^ and the type strains of *E. hormaechei* subsp. *oharae*, *E. hormaechei* subsp. *steigerwaltii*, and *E. xiangfangensis* ranges from 66.5% to 66.9% (Table 2), lower than the 70% cutoff to define a bacterial species. Therefore, *E. hormaechei* subsp. *hoffmannii* is a novel *Enterobacter* species rather than a subspecies of any known *Enterobacter* species, and we propose the species name as *Enterobacter hoffmannii*.

### *Enterobacter timonensis* should be removed to a novel genus with the proposed name *Pseudenterobacter*.

**FIG 1:**
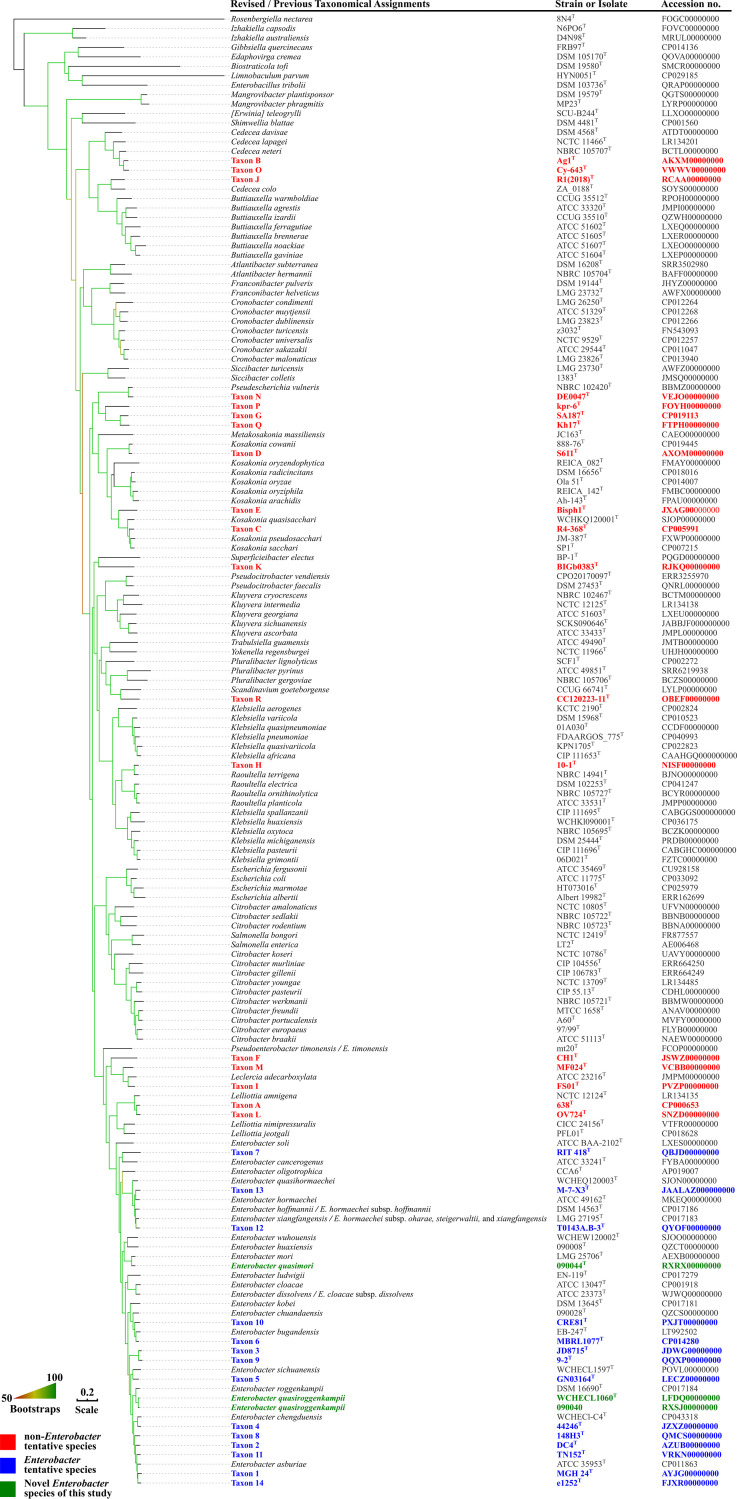
A phylogenetic tree based on the concatenated nucleotide sequence of core genes of *Enterobacter quasimori* strain 090044^T^, *Enterobacter quasiroggenkampii* strains WCHECL1060^T^ and 090040, non-*Enterobacter* tentative taxons A to T, *Enterobacter* tentative taxons 1 to 14, and type strains of the family *Enterobacteriaceae* (listed in Data Set S1). Strains and their nucleotide accession numbers are listed alongside the species names. For species and subspecies with names that need to be revised as suggested in this study, the revised names are shown first, and the current names are shown after the slash. The tree was inferred using the maximum likelihood method under the GTRGAMMA model with a 1,000-bootstrap test, and branches with support over 50% are indicated by gradients. Bar, value indicates the nucleotide substitutions per site.

**FIG 2:**
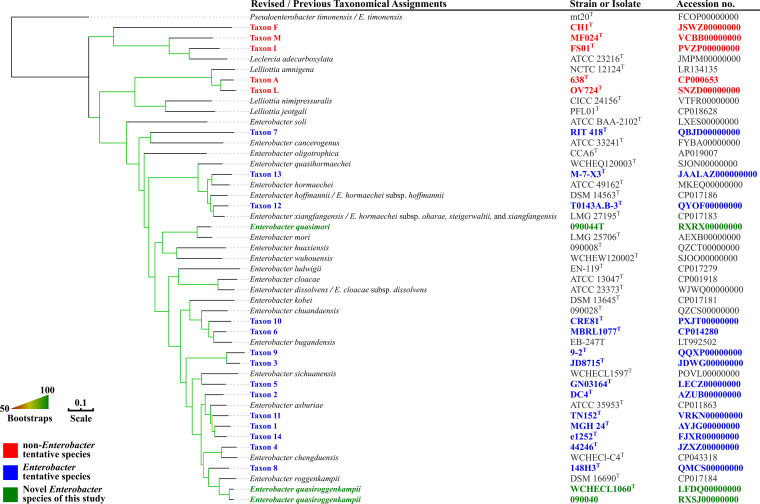
A more precise phylogenetic tree based on the concatenated nucleotide sequence of core genes of tentative taxons and type strains of genera *Enterobacter*, *Leclercia*, and *Lelliottia* (listed in Tables 5 and 7 and Data Set S1). Strains and their nucleotide accession numbers are listed alongside the names of species. For species and subspecies with names that need to be revised as suggested in this study, the revised names are shown first, and the current names are shown after the slash. The tree was inferred using the maximum likelihood method under the GTRGAMMA model with a 1,000-bootstrap test, and branches with support over 50% are indicated by gradients. Bar, value indicates the nucleotide substitutions per site.

The core gene-based phylogenomic tree of the family *Enterobacteriaceae* (Fig. 1) and that of the genus *Enterobacter* and closely related genera (Fig. 2) demonstrated that *E. timonensis* forms an independent branch, which is well separated from all other *Enterobacter* species by species of the genera *Leclercia* and *Lelliottia.* The ANI values between the type strain of *E. timonensis* and those of all other *Enterobacter* species are <85% (82.03 to 83.78%, Table S1), while the values between type strains of other *Enterobacter* species are >85%. Correspondingly, the isDDH values between the type strain of *E. timonensis* and those of all other *Enterobacter* species are <30% (24.7 to 26.3%, Table S1), while the values between type strains of other *Enterobacter* species are >30%. The above findings suggest that *E. timonensis* does not belong to the genus *Enterobacter*. The ANI and isDDH values for the type strain of *E. timonensis* and those of *Leclercia* and *Lelliottia* species are <85% and <30%, respectively. The phylogenomic trees (Fig. 1 and 2) demonstrated that *E. timonensis* is also distinct from *Leclercia* and *Lelliottia* species. Therefore, it is evident that *E. timonensis* does not belong to the genus *Leclercia* nor *Lelliottia* but to a novel genus. As it is closely related to *Enterobacter*, we propose the genus name *Pseudenterobacter* (Pseud.en.te.ro.bac′ter. Gr. adj. pseudês false; N.L. masc. n. *Enterobacter* a bacterial generic name; N.L. fem. n. *Pseudenterobacter*, a genus falsely [or incorrectly] classified in *Enterobacter*). *E. timonensis* should therefore be renamed *Pseudenterobacter timonensis*.

10.1128/mSystems.00527-20.8 DATA SET S1 Type or reference strains of species within the family *Enterobacteriaceae*. Download Data Set S1, XLSX file, 0.02 MB. Copyright © 2020 Wu et al. 2020 Wu et al. This content is distributed under the terms of the Creative Commons Attribution 4.0 International license.

### Two *Enterobacter* strains from blood represent a novel species, named *Enterobacter quasiroggenkampii* sp. nov.

Strains WCHECL1060^T^ and 090040 were both identified as E. cloacae by Vitek II. The two strains had very different genomic fingerprints obtained by macrorestriction analysis (see Fig. S2 in the supplemental material). The 16S rRNA gene sequence of the two strains shared 99.61% identity (6 bases mismatch) and was 99% identical to those of type strains of a few *Enterobacter* species including *E. asburiae*, E. cloacae, *E. hormaechei*, *E. kobei*, and *E. ludwigii*.

10.1128/mSystems.00527-20.3 FIG S2 Genomic DNA restriction patterns of strain WCHECL1060^T^ and 090040. Both strains were subjected to XbaI macrorestriction analysis, which was performed as described previously (13). M, Lambda Ladder PFG marker N0340S (NEB). 1060, WCHECL1060^T^. The macrorestriction analysis for both strains was performed twice. Download FIG S2, TIF file, 0.7 MB. Copyright © 2020 Wu et al. 2020 Wu et al. This content is distributed under the terms of the Creative Commons Attribution 4.0 International license.

The draft whole-genome sequence of strain WCHECL1060^T^ has been reported by us before (22), and its 4.8-Mb draft genome was assembled from 1.7 gigabases into 21 contigs ≥200 bp in length (*N*_50_, 714,400 bp) with a 55.68% GC content. For strain 090040, 4,788,302 reads and 1.73 gigabases were generated, which were assembled into a 4.9-Mb draft genome containing 30 contigs ≥200 bp in length (*N*_50_, 515,146 bp) with a 55.69% GC content. No contamination was identified for the genomes of WCHECL1060^T^ and 090040. The ANI value between strains WCHECL1060^T^ and 090040 was 98.4% (Table 3). In contrast, the ANI values between the two strains and type strains of all known *Enterobacter* species were <96% and the highest value (95.37%/95.30%, respectively) was seen with *E. roggenkampii* DSM 16690^T^ (Table 3 and Table S1). The isDDH value between strains WCHECL1060^T^ and 090040 was 88% (Table 3), whereas isDDH values between the two strains and type strains of all known *Enterobacter* species were 64.8%/65.2%, respectively (with *E. roggenkampii* DSM 16690^T^), or lower (Table 3 and Table S1), which were below the 70% cutoff to define a bacterial species. Therefore, the ANI and isDDH analyses clearly suggest that the two strains represent a novel species of the genus *Enterobacter*.

**TABLE 3:** Average nucleotide identity, *in silico* DNA-DNA hybridization, and percentage of conserved proteins values between strains WCHECL1060^T^, 090040, and 090044^T^ and the type strain of species belonging to the genus *Enterobacter*

Species and/or strain	Accession no.	ANI/isDDH, %, for strain:		
		WCHECL1060^T^	090040	090044^T^
*E. asburiae* ATCC 35953^T^	CP011863	93.25/51.7	93.01/51.8	90.07/40.9
*E. bugandensis* EB-247^T^	FYBI00000000	91.02/43.1	90.62/43.2	88.95/38.0
*E. cancerogenus* ATCC 33241^T^	ERR1854846	86.39/31.5	85.70/31.6	86.33/32.3
*E. chengduensis* WCHECL-C4^T^	MTSO00000000	92.19/49.3	92.02/49.3	89.10/38.7
*E. chuandaensis* 090028^T^	QZCS00000000	90.49/42.7	90.60/42.9	89.25/38.5
*E. cloacae* ATCC 13047^T^	CP001918	88.28/35.8	87.53/35.8	87.30/34.4
*E. dissolvens* ATCC 23373^T^	WJWQ00000000	87.92/35.9	87.94/35.9	87.52/34.5
*E. hoffmannii* DSM 14563^T^	CP017186	86.91/33.5	86.93/33.7	87.76/34.8
*E. hormaechei* ATCC 49162^T^	MKEQ00000000	87.51/33,7	86.90/33.8	87.77/35.0
*E. huaxiensis* 090008^T^	QZCT00000000	87.36/34.5	87.20/34.6	88.72/37.6
*E. kobei* DSM 13645^T^	CP011863	90.26/40.6	89.72/40.7	88.19/36.1
*E. ludwigii* EN-119^T^	CP017279	88.03/34.9	87.70/35.0	86.91/33.0
*E. mori* LMG 25706^T^	AEXB00000000	88.98/37.3	88.15/37.4	95.32/66.8
*E. oligotrophica* CCA6^T^	AP019007	84.62/28.4	84.58/28.4	87.81/34.9
*E. quasihormaechei* WCHEs120003^T^	SJON00000000	86.97/33.6	87.80/33.8	87.63/34.9
*E. roggenkampii* DSM 16690^T^	CP017184	95.37/64.8	95.30/65.2	89.62/39.8
*E. sichuanensis* WCHECL1597^T^	POVL00000000	91.10/44.7	90.82/44.8	88.07/36.4
*E. soli* ATCC BAA-2102^T^	LXES00000000	85.93/30.8	85.21/30.7	85.70/31.0
*E. wuhouensis* WCHEs120002^T^	SJOO00000000	87.62/35.7	88.38/35.8	88.91/38.1
*E. xiangfangensis* LMG 27195^T^	CP017183	87.54/34.0	87.09/34.0	87.74/35.0
*Pseudenterobacter timonensis* mt20^T^	FCOP00000000	82.03/25.9	82.20/26.0	82.41/25.8
WCHECL1060^T^	LFDQ00000000		98.40/88.0	89.68/40.2
090044^T^	RXSJ00000000	89.57/40.2	89.65/40.3	

Biochemical characteristics between strains WCHECL1060^T^ and 090040 and type strains of other *Enterobacter* species are shown in Table 4. For both strains, growth occurs at 4 to 37°C with optimal growth at 35 and 37°C, but not at 45 or 50°C. Cells grow at 35°C in the presence of 0 to 9% (wt/vol) NaCl in tryptic soy broth (TSB). Both strains were positive for the catalase test but negative for oxidase activity. Cells of the two strains are Gram negative, motile, non-spore-forming, facultatively anaerobic, and rod shaped. Colonies are circular, white, translucent, raised, and smooth after 24 h of incubation at 35°C on nutrient agar. Acid is produced from glycerol, l-arabinose, d-ribose, d-xylose, d-galactose, d-glucose, sucrose, melibiose, amygdalin, d-fructose, d-mannose, l-rhamnose, inositol, d-mannitol, d-sorbitol, potassium 2-ketogluconate, and methyl-α-d-glucopyranoside but not erythritol, l-xylose, d-adonitol, d-arabinose, potassium gluconate, and methyl-α-d-mannopyranoside. Both strains have a positive reaction for β-galactosidase, arginine dihydrolase, and ornithine decarboxylase but are negative for lysine decarboxylase, deaminase, and gelatinase. Both are also negative for urease activity and indole production but positive for the Voges-Proskauer reaction. Both strains can utilize citrate but do not produce H_2_S. The two strains can be differentiated from all other *Enterobacter* species by their ability to ferment inositol, d-sorbitol, and melibiose but not potassium gluconate, l-fucose, and methyl-α-d-mannopyranoside.

**TABLE 4:** Biochemical characteristics of strains WCHECL1060^T^ and 090040 and type strains of other *Enterobacter* species^*a*^

Characteristic	Result for species:																				
	1	2	3	4	5	6	7	8	9	10	11	12	13	14	15	16	17	18	19	20	21
β-Galactosidase	+	+	+	+	+	+	+	+	+	+	+	+	+	+	+	+	+	+	−	+	+
Arginine dihydrolase	+	+	+	+	+	+	+	+	+	+	+	+	+	+	−	+	+	+	+	+	−
Lysine decarboxylase	−	−	−	−	−	−	−	−	−	−	−	−	−	−	−	−	−	−	−	+	+
Ornithine decarboxylase	+	+	+	+	+	−	+	+	+	+	+	+	+	+	+	+	−	+	−	+	+
Citrate utilization	+	+	+	+	+	+	+	+	+	+	+	+	+	+	+	+	(+)	+	+	+	−
H_2_S production	−	−	−	−	−	−	−	−	−	−	−	−	−	−	−	−	−	−	−	−	−
Urea hydrolysis	−	−	−	−	−	−	−	−	−	−	−	−	−	−	−	−	−	−	−	−	−
Deaminase	−	−	−	−	−	−	−	−	+	−	−	−	−	−	−	−	−	−	−	−	−
Indole production	−	−	−	−	−	−	−	−	−	−	−	−	−	−	−	−	−	−	−	−	+
Voges-Proskauer reaction	+	+	+	+	+	+	+	−	W	+	+	+	+	+	−	−	+	+	+	−	−
Gelatinase	−	−	−	−	−	−	−	−	−	−	−	−	−	−	−	−	−	−	−	−	−
d-Glucose	+	+	+	+	+	+	+	+	+	+	+	+	+	+	+	+	+	+	+	+	+
d-Mannitol	+	+	+	+	+	+	−	+	+	+	+	+	+	+	+	+	+	+	+	+	+
Inositol	+	+	−	−	−	−	+	W	−	−	+	−	+	−	−	−	−	+	−	−	−
d-Sorbitol	+	+	−	−	+	−	+	+	+	+	+	+	+	−	+	−	+	+	+	+	+
l-Rhamnose	+	+	+	+	+	+	−	+	+	+	+	+	+	+	−	+	+	+	+	+	+
Sucrose	+	+	+	+	+	+	+	+	+	+	+	+	+	−	+	+	+	+	−	−	−
Melibiose	+	+	+	+	+	−	+	+	+	+	+	+	+	−	−	−	+	+	+	−	+
Amygdalin	+	+	+	+	+	+	+	+	+	+	+	+	+	+	+	+	+	+	+	+	−
Arabinose	+	+	+	+	+	+	+	+	+	+	+	+	+	+	+	+	+	+	+	+	+
Potassium gluconate	−	−	−	+	−	+	−	+	ND	+	−	−	+	−	+	+	−	ND	ND	ND	ND
Methyl-α-d-mannopyranoside	−	−	−	−	−	−	−	−	ND	+	+	−	+	−	+	W	+	ND	ND	−	ND
l-Fucose	−	−	−	−	−	−	−	−	ND	−	V	−	V	+	−	+	−	+	ND	+	ND
d-Arabitol	−	−	−	−	+	−	−	−	−	(−)	+	−	−	−	−	(−)	−	−	ND	−	ND
Dulcitol	−	+	−	−	−	−	−	−	ND	W	−	+	−	−	−	+	−	+	ND	+	ND
d-Turanose	−	+	ND	ND	−	−	−	+	ND	−	+	−	−	−	W	+	−	W	ND	−	ND
Motility	+	+	+	+	+	+	−	+	+	+	+	+	+	+	−	+	+	+	ND	ND	ND

a Species: 1, *E. quasiroggenkampii*; 2, *E. quasimori*; 3, *E. wuhouensis*; 4, *E. quasihormaechei*; 5, *E. huaxiensis*; 6, *E. chuandaensis*; 7, *E. sichuanensis*; 8, *E. chengduensis*; 9, *E. soli*; 10, E. cloacae; 11, *E. mori*; 12, *E. bugandensis*; 13, *E. ludwigii*; 14, *E. cancerogenus*; 15, *E. asburiae*; 16, *E. hormaechei*; 17, *E. xiangfangensis*; 18, *E. kobei*; 19, *E. timonensis*; 20, *E. oligotrophica*; 21, E. coli ATCC 25922. Data for species other than *E. quasiroggenkampii* and *E. quasimori* are from references 14, 19, 24, 40, 42, and 45 to 50. Results for E. coli ATCC 25922 are consistent with the results listed in The Bacterial Diversity Metadatabase at http://bacdive.dsmz.de/index.php?search=atcc+25922&submit=Search. +, 90 to 100% positive reaction; (+), 80 to 90% positive; −, 0 to 10% positive reaction; (−), 10 to 20% positive; W, weakly positive; V, varied; ND, not determined.

The comparison of the fatty acid profiles of the strains WCHECL1060^T^ and 090040 and type strains of other *Enterobacter* species are shown in Table S2 in the supplemental material. Although the proportions of the fatty acids were slightly different, the major cellular fatty acids of strains WCHECL1060^T^ and 090040 were C_16:0_, C_17:0_ cyclo, and C_18:1ω7c_, which were consistent with those of other *Enterobacter* species. The antimicrobial susceptibility profiles and antimicrobial resistance genes of the two strains are described in the supplemental material (Text S1 and Table S3).

10.1128/mSystems.00527-20.1 TEXT S1 Antimicrobial susceptibility and resistance genes. Download Text S1, DOCX file, 0.02 MB. Copyright © 2020 Wu et al. 2020 Wu et al. This content is distributed under the terms of the Creative Commons Attribution 4.0 International license.

10.1128/mSystems.00527-20.5 TABLE S2 Fatty acid profiles of the strains *E. quasiroggenkampii* WCHECL1060^T^, *E. quasiroggenkampii* 090040, and *E. quasimori* 090044^T^ and other type strains of *Enterobacter* species. Download Table S2, DOCX file, 0.02 MB. Copyright © 2020 Wu et al. 2020 Wu et al. This content is distributed under the terms of the Creative Commons Attribution 4.0 International license.

10.1128/mSystems.00527-20.6 TABLE S3 Antimicrobial susceptibility of strains WCHECL1060^T^, 090040, and 090044^T^. Download Table S3, DOCX file, 0.01 MB. Copyright © 2020 Wu et al. 2020 Wu et al. This content is distributed under the terms of the Creative Commons Attribution 4.0 International license.

The results presented here indicate that two strains represent a novel species within the genus *Enterobacter*, which is clearly distinct from all known *Enterobacter* species. As it is most closely related to *E. roggenkampii* in whole-genome analysis, we propose the name *Enterobacter quasiroggenkampii* sp. nov. (qua.si.rog.gen.kamp.i; L. adv. *quasi* nearly, almost; N.L. gen. n. *roggenkampii* of Roggenkamp, and a specific epithet in the genus *Enterobacter*; N.L. gen. n. *quasiroggenkampii* almost *roggenkampii*) for this species with WCHECL1060^T^ (= GDMCC 1.1742^T^ = KCTC 52992^T^) as the type strain.

### An *Enterobacter* strain from blood represents another novel species, named *Enterobacter quasimori* sp. nov.

Strain 090044^T^ was identified as E. cloacae by Vitek II. The 16S rRNA gene sequence of the strain was 99% identical to those of type strains of a few *Enterobacter* species including *E. asburiae*, *E. bugandensis*, *E. hormaechei*, *E. kobei*, and *E. ludwigii*. Whole-genome sequencing for strain 090044^T^ generated 4,498,239 reads and 1.35 gigabases, which were assembled into a 4.71-Mb draft genome containing 53 contigs ≥200 bp in length (*N*_50_, 291,547 bp) with a 55.76% GC content. No contamination was identified. The ANI values between strain 090044^T^ and type strains of all known *Enterobacter* species and WCHECL1060^T^ were <96%, and the highest value (95.32%) was seen with *E. mori* LMG 25706^T^ (Table 3 and Table S1). The isDDH values between strain 090044^T^ and type strains of all known *Enterobacter* species and WCHECL1060^T^ were <70%, and the highest value (66.8%) was seen with *E. mori* LMG 25706^T^ (Table 3 and Table S1). Therefore, based on the ANI and isDDH analyses, it is evident that the strain represents a novel species of the genus *Enterobacter*.

For strain 090044^T^, growth occurs at 4 to 37°C with optimal growth at 35 and 37°C, but not at 45 or 50°C. Cells grow at 35°C in the presence of 0 to 9% (wt/vol) NaCl in TSB. They are positive for the catalase test but negative for oxidase activity. Cells of strain 090044^T^ are Gram negative, motile, non-spore-forming, facultatively anaerobic, and rod shaped. Colonies are circular, white, translucent, raised, and smooth after 24 h of incubation at 35°C on nutrient agar. Acid is produced from glycerol, l-arabinose, d-ribose, d-xylose, d-galactose, d-glucose, sucrose, melibiose, amygdalin, d-fructose, d-mannose, l-rhamnose, inositol, d-mannitol, d-sorbitol, dulcitol, d-turanose, potassium 2-ketogluconate, and methyl-α-d-glucopyranoside but not erythritol, l-xylose, d-adonitol, d-arabinose, potassium gluconate, and methyl-α-d-mannopyranoside. Strain 090044^T^ has a positive reaction for β-galactosidase, arginine dihydrolase, and ornithine decarboxylase but is negative for lysine decarboxylase, deaminase, and gelatinase. It is also negative for urease activity and indole production but positive for the Voges-Proskauer reaction. It can utilize citrate but does not produce H_2_S. It is catalase positive and oxidase negative. Strain 090044^T^ can be differentiated from other *Enterobacter* species and WCHECL1060^T^ by its ability to ferment inositol, d-sorbitol, dulcitol, d-turanose, and melibiose but not potassium gluconate, l-fucose, and methyl-α-d-mannopyranoside. The major cellular fatty acids of strain 090044^T^ were C_16:0_, C_17:0_ cyclo, and C_18:1ω7c_, which were consistent with those of other *Enterobacter* species (Table S2). The antimicrobial susceptibility profile and antimicrobial resistance genes of the strain are described in the supplemental material (Text S1 and Table S3).

The results presented here indicate that strain 090044^T^ represents a novel species within the genus *Enterobacter*. As it is most closely related to *E. quasimori* in whole-genome analysis, we propose the name *Enterobacter quasimori* sp. nov. (qua.si.mo.ri; L. adv. *quasi* nearly, almost; N.L. gen. n. *mori* of Zhu, and a specific epithet in the genus *Enterobacter*; N.L. gen. n. *quasimori* almost *mori*) for this species with 090044^T^ (= GDMCC 1.1735^T^ = JCM 33940^T^) as the type strain.

### Most *Enterobacter* genomes in GenBank need to be curated for precise species identification.

Based on the above findings, the taxonomy of *Enterobacter* should be updated to comprise 22 species at present (Table 5). There were 1,997 *Enterobacter* strains with genomes deposited in GenBank, and the species identification is required to be curated for most (*n* = 1,542, 77.2%) of these strains in four scenarios. First, among 1,997 *Enterobacter* strains with genomes deposited in GenBank, 1,960 were indeed *Enterobacter* strains but 37 did not belong to the genus *Enterobacter*. Five strains did not even belong to the family *Enterobacteriaceae* but rather belonged to the genus *Pantoea* of the family *Erwiniaceae* (*n* = 4) or the genus *Serratia* of the family *Yersiniaceae* (*n* = 1; Data Set S2). Thirty strains belonged to other species of the family *Enterobacteriaceae*, among which 7 belonged to known species including Atlantibacter subterranea, Citrobacter portucalensis, Escherichia coli, Klebsiella aerogenes, and Klebsiella pneumoniae, while 23 strains could not be assigned to known species. We found the 23 strains actually belonged to 18 novel unnamed species, which are tentatively assigned to taxons A to R here (Table S4). Two belong to *E. timonensis*, which should be removed to the genus *Pseudenterobacter*. Second, of the 1,960 *Enterobacter* strains, 155 strains were only labeled as *Enterobacter* spp. (*n* = 117), E. cloacae complex (*n* = 34), or *Enterobacter* genomosp. (*n* = 4) but were not assigned to the species level (Data Set S2). Third, species were misidentified for 481 *Enterobacter* strains, most (*n* = 460) of which were labeled as E. cloacae but actually belonged to other *Enterobacter* species. Fourth, there were 869 strains whose species identification needs to be updated according to the findings in this study. In particular, only 80 (14.8%) out of the 540 genomes labeled as E. cloacae actually belonged to the species, while only 13 (1.5%) out of the 880 genomes labeled as *E. hormaechei* (*n* = 509) or one of its subspecies (*n* = 371) were truly *E. hormaechei*.

**TABLE 5:** Updated classification and nomenclature of the genus *Enterobacter*

Species (*n* = 22)	Type strain	Accession no.
*Enterobacter asburiae*^*a*^	JCM 6051	CP011863
*Enterobacter bugandensis*	EB-247	FYBI00000000
*Enterobacter cancerogenus*	ATCC 35316	ERR1854846
*Enterobacter chengduensis*	WCHECl-C4	MTSO00000000
*Enterobacter chuandaensis*	090028	QZCS00000000
*Enterobacter cloacae*	ATCC 13047	CP001918
*Enterobacter dissolvens*^*b*^	ATCC 23373	WJWQ00000000
*Enterobacter hoffmannii*^*c*^	DSM 14563	CP017186
*Enterobacter hormaechei*	ATCC 49162	MKEQ00000000
*Enterobacter huaxiensis*	090008	QZCT00000000
*Enterobacter kobei*	ATCC BAA-260	CP017181
*Enterobacter ludwigii*	EN-119	CP017279
*Enterobacter mori*^*d*^	LMG 25706	GL890773
*Enterobacter oligotrophica*	CCA6	AP019007
*Enterobacter quasihormaechei*	WCHEs120003	SJON00000000
*Enterobacter quasimori*	090044	RXRX00000000
*Enterobacter quasiroggenkampii*	WCHECL1060	LFDQ00000000
*Enterobacter roggenkampii*	DSM 16690	CP017184
*Enterobacter sichuanensis*	WCHECl1597	POVL00000000
*Enterobacter soli*	ATCC BAA-2102	LXES00000000
*Enterobacter wuhouensis*	WCHEs120002	SJOO00000000
*Enterobacter xiangfangensis*^*e*^	LMG 27195	CP017183

a Enterobacter muelleri is a later synonym of Enterobacter asburiae (42).

b Previously known as Enterobacter cloacae subsp. *dissolvens*.

c Previously known as Enterobacter hormaechei subsp. *hoffmannii*.

d Enterobacter tabaci is a later synonym of Enterobacter mori (15).

e Enterobacter hormaechei subsp. *oharae* and Enterobacter hormaechei subsp. *steigerwaltii* are later synonyms of Enterobacter xiangfangensis.

10.1128/mSystems.00527-20.7 TABLE S4 Tentative taxon assignations for new, unnamed non-*Enterobacter* species of the family *Enterobacteriaceae*. Download Table S4, DOCX file, 0.01 MB. Copyright © 2020 Wu et al. 2020 Wu et al. This content is distributed under the terms of the Creative Commons Attribution 4.0 International license.

10.1128/mSystems.00527-20.9 DATA SET S2 *Enterobacter* strains with genome sequences available in GenBank and the precise species identification. Download Data Set S2, XLSX file, 0.2 MB. Copyright © 2020 Wu et al. 2020 Wu et al. This content is distributed under the terms of the Creative Commons Attribution 4.0 International license.

After curation of precise species identification, among the 1,960 *Enterobacter* strains, half (*n* = 994, 50.7%) actually belonged to *E. xiangfangensis*, while *E. hoffmannii* is the second most common species with 287 strains (14.7%; Table 6), followed by *E. asburiae* (*n* = 116, 5.9%) and *E. roggenkampii* (*n* = 112, 5.7%). However, there were 60 (3.1%) strains that could not be assigned to any known *Enterobacter* species. Instead, the 60 strains can be assigned to 14 potentially novel *Enterobacter* species, which are unnamed as they have not been characterized by phenotype methods. The 14 potentially novel *Enterobacter* species were assigned taxons 1 to 14 here (Table 7). There were 1,496 strains from human specimens. Among strains from human, *E. xiangfangensis* was still the most common species (805/1,496, 53.8%; Table 6) and *E. hoffmannii* was the second most common (251/1,496, 17.2%). Although the selection of bacterial strains is usually biased for genome sequencing, the common identification of the two *Enterobacter* species from human specimens is unlikely to be a coincidence. The reasons why isolates of the two *Enterobacter* species are commonly recovered from human specimens warrant further studies.

**TABLE 6:** Species distribution of 1,960 *Enterobacter* strains with genome sequences available in GenBank

Proposed species	No., all sources	No., human strains
*Enterobacter asburiae*	116	78
*Enterobacter bugandensis*	55	42
*Enterobacter cancerogenus*	14	3
*Enterobacter chengduensis*	5	5
*Enterobacter chuandaensis*	2	1
*Enterobacter cloacae*	86	60
*Enterobacter dissolvens*	15	7
*Enterobacter hoffmannii*	287	251
*Enterobacter hormaechei*	13	8
*Enterobacter huaxiensis*	2	2
*Enterobacter kobei*	97	72
*Enterobacter ludwigii*	55	28
*Enterobacter mori*	9	5
*Enterobacter oligotrophica*	1	6
*Enterobacter quasihormaechei*	9	5
*Enterobacter quasiroggenkampii*	8	0
*Enterobacter roggenkampii*	112	75
*Enterobacter sichuanensis*	15	12
*Enterobacter soli*	4	0
*Enterobacter wuhouensis*	1	1
*Enterobacter xiangfangensis*	994	805
Taxon 1	2	0
Taxon 2	10	3
Taxon 3	8	2
Taxon 4	14	11
Taxon 5	4	4
Taxon 6	2	1
Taxon 7	1	0
Taxon 8	8	4
Taxon 9	2	0
Taxon 10	3	2
Taxon 11	1	0
Taxon 12	1	1
Taxon 13	2	0
Taxon 14	2	2
Total	1,960	1,496

**TABLE 7:** Tentative taxon assignations for novel, unnamed *Enterobacter* species

Taxon	Accession no.	Reference strain^*a*^	Closest species	ANI, %	DDH, %
1	AYJG00000000	MGH 24	*E. asburiae*	95.403	63.70
2	AZUB00000000	DC4	*E. quasiroggenkampii*	94.884	58.70
3	JDWG00000000	JD8715	*E. asburiae*	90.937	42.00
4	JZXZ00000000	44246	*E. chengduensis*	95.521	64.40
5	LECZ00000000	GN03164	*E. asburiae*	92.904	49.70
6	CP014280	MBRL1077	*E. bugandensis*	95.532	63.70
7	QBJD00000000	RIT 418	*E. wuhouensis*	87.641	32.10
8	QMCS00000000	148H3	*E. quasiroggenkampii*	94.463	56.50
9	QQXP00000000	9-2	*E. asburiae*	90.268	40.30
10	PXJT00000000	CRE81	*E. bugandensis*	94.295	55.90
11	VRKN00000000	TN152	*E. asburiae*	95.255	62.50
12	QYOF00000000	T0143A.B-3	*E. xiangfangensis*	95.989	66.80
13	JAALAZ000000000	M-7-X3	*E. xiangfangensis*	94.957	60.10
14	FJXR00000000	e1252	*E. asburiae*	95.660	65.00

a The strain with genome sequence deposited in GenBank at the earliest date was selected as the reference strain.

## DISCUSSION

In this study, we first updated the taxonomy of the genus *Enterobacter* and modified the taxonomic assignments for *E. timonensis* and the subspecies of E. cloacae and *E. hormaechei* by genome analyses and also reported two novel species, which were characterized by both genome- and phenotype-based methods. We then applied the updated taxonomy assignments to curate genome sequences deposited in GenBank with the label of *Enterobacter* and found that the species identification of most *Enterobacter* strains with genome sequences available needed to be corrected.

We found that all subspecies assignments in the genus *Enterobacter* were incorrect and their use should be discontinued. Genetic clustering of the *hsp60* (a housekeeping gene) sequence has been used as the premise for assigning *E. hormaechei* subsp. *hormaechei*, *E. hormaechei* subsp. *oharae*, and *E. hormaechei* subsp. *steigerwaltii* (16, 17). However, determining taxonomic assignment using a single-gene-based approach has omitted valuable information available from the rest of the genome and potentially led to unreliable conclusions about taxonomic positions. Such subspecies assignment should be rigorously reexamined based on analysis of whole-genome sequences. Indeed, on the basis of whole-genome-based analysis, it becomes evident that the subspecies of *E. hormaechei* actually belong to three species. *E. xiangfangensis* is not a subspecies of *E. hormaechei* but an independent species, while *E. hormaechei* subsp. *steigerwaltii* and *E. hormaechei* subsp. *oharae* belong to the same species as *E. xiangfangensis*. *E. hormaechei* subsp. *hoffmannii* is a novel species, *E. hoffmannii*. Whole-genome-based analysis also reveals that E. cloacae subsp. *dissolvens* is actually a species, *E. dissolvens*, rather than a subspecies of E. cloacae. The above findings also highlight that the assignment of subspecies should be prudent as there is no general guideline for defining subspecies using genome data (23) and subspecies assignment requires rigorous studies. These studies should include large-scale properly designed investigations on clinical significance such as host specificity of these bacteria to examine the rationale why subspecies should be created and separately recognized (23) and to avoid unnecessary confusion or even chaos.

We also found that most genomes labeled as E. cloacae and *E. hormaechei* are not correctly identified to the species level. The incorrect identification may be due to different reasons. Of note, the ≥95% ANI cutoff alone is widely used for species assignment, but such a cutoff is unable to resolve closely related species (24). Previous studies have corroborated that the stringent ≥96% ANI cutoff is more accurate with better correlation with the 70% DDH cutoff (11) but also highlight that species assignment based on a single algorithm may not be robust. In this study, we employed both ANI with a ≥96% ANI cutoff and isDDH for robust species assignment. In addition, for E. cloacae, phenotype-based tests used in clinical microbiology laboratories commonly identify *Enterobacter* clinical isolates as E. cloacae as evidenced by the misidentification of strains WCHECL1060^T^, 090040, and 090044^T^ by Vitek II. In contrast, for *E. hormaechei*, incorrect identification was mainly due to incorrect subspecies assignments as discussed above. This highlights that updated and curated taxonomic assignments are the premise of correct and precise species identification. We suggest that future studies on *Enterobacter* need to consider the correct species and subspecies identification to provide robust results while avoiding misleading information.

We report two novel *Enterobacter* species here and found that there were 14 tentative novel *Enterobacter* species and 18 tentative non-*Enterobacter* species of the family *Enterobacteriaceae*, which are clearly listed in the study. This invites more studies on these tentative species by both genome- and phenotype-based methods to establish their species status and to propose appropriate species names. Such studies will further reveal the complicated taxonomy of *Enterobacter*, a genus of bacterial species with clinical significance.

### Conclusions.

All subspecies assignments in the genus *Enterobacter* were incorrect, and their use should be discontinued. E. cloacae subsp. *dissolvens* is a species and should be renamed *E. dissolvens*. *E. xiangfangensis* is not a subspecies of *E. hormaechei*, while *E. hormaechei* subsp. *oharae* and *E. hormaechei* subsp. *steigerwaltii* are not subspecies of *E. hormaechei* but belong to the same species of *E. xiangfangensis*. *E. hormaechei* subsp. *hoffmannii* is a species and should be renamed as *E. hoffmannii*. *E. timonensis* should be removed to *Pseudenterobacter*, a novel genus. Two novel *Enterobacter* species, *E. quasiroggenkampii* and *E. quasimori*, were identified. *E. quasiroggenkampii* can be distinguished from all known *Enterobacter* species by its ability to ferment inositol, d-sorbitol, and melibiose but not potassium gluconate, l-fucose, and methyl-α-d-mannopyranoside. *E. quasimori* can be distinguished from all known *Enterobacter* species by its ability to ferment inositol, d-sorbitol, dulcitol, d-turanose, and melibiose but not potassium gluconate, l-fucose, and methyl-α-d-mannopyranoside. The species identifications for most *Enterobacter* strains with genomes deposited in GenBank are required to be curated. The most common *Enterobacter* species seen in clinical samples appears to be *E. xiangfangensis*. Fourteen novel tentative *Enterobacter* genome species were also found and warrant further phenotype-based characterizations.

## MATERIALS AND METHODS

### Strain and initial species identification.

The type strain of E. cloacae subsp. *dissolvens* ATCC 23373^T^ was obtained from the Guangdong Microbial Culture Collection Center (http://www.gdmcc.net/). Three nonduplicated clinical strains, WCHECL1060^T^, 090040, and 090044^T^, were all recovered from the blood culture of three different patients with fever as part of routine patient care at West China Hospital of Sichuan University, Chengdu, China, in 2014 or 2016. This study has been approved by the Ethical Committee of West China Hospital, and the informed consent was waived as this study was to retrospectively characterize bacterial strains that were collected as part of routine patient care.

Initial species identification was performed using the Vitek II automated system (bioMérieux, Marcy l'Etoile, France). The 16S rRNA gene sequences of the three strains were obtained as described previously (25) and were compared using a pairwise nucleotide sequence alignment tool (https://www.ezbiocloud.net/tools/pairAlign) using Myers and Miller’s algorithm (26). As strains WCHECL1060^T^ and 090040 belonged to the same species, they were subjected to pulsed-field gel electrophoresis by XbaI macrorestriction, which was performed as described previously (27), to determine their clonal relatedness.

### Whole-genome sequencing.

We have reported the draft genome of strain WCHECL1060^T^ before (22). Genomic DNA of E. cloacae subsp. *dissolvens* ATCC 23373^T^, strain 090040, and strain 090044^T^ was prepared using the QIAamp DNA minikit (Qiagen, Hilden, Germany), and DNA sequencing libraries were prepared using the NEBNext Ultra II DNA Library Prep kit for Illumina (NEB, Ipswich, MA, USA). Whole-genome sequencing was performed using the HiSeq 2500 Sequencer (Illumina, San Diego, CA, USA) with the 150-bp paired-end protocol and about 200× coverage. Reads were trimmed using Trimmomatic v0.39 (28) under the default setting and were then assembled into contigs using SPAdes v3.11.1 (29) under careful mode. Genome completeness and contamination were examined using CheckM v1.0.18 (30). The genome sequences were reported following recommendations of standards for describing a new taxonomy (23).

### Phylogenetic analysis of the genus *Enterobacter* based on core genes.

Whole-genome sequences of the type strains of all species and subspecies within the genus *Enterobacter* and all other species of the family *Enterobacteriaceae* (listed in Data Set S1 in the supplemental material) were retrieved from the NCBI database. A core genome phylogenetic tree based on concatenated sequences of core genes was constructed as described previously (31). Prokka v1.12 (32) was used to annotate these genome sequences, and orthologues of these strains were identified using OrthoFinder v2.26 (33) to represent the core genome of these *Enterobacteriaceae* strains. The gene sequences were aligned and concatenated using MAFFT v7.313 (34) and AMAS v0.98 (35), which were then used to infer a phylogenomic tree using RAxML v8.2.12 (36) with GTR model plus gamma distribution and a 1,000-bootstrap test.

### Determination of overall genome relatedness.

The pairwise average nucleotide identity (ANI) and *in silico* DNA-DNA hybridization (isDDH) between strains ATCC 23373^T^, WCHECL1060^T^, 090040, and 090044^T^ and the type strain of *Enterobacter* species and subspecies were determined using the JSpecies program based on BLAST (jspecies.ribohost.com) (37) and GGDC (formula 2) (10), respectively. A ≥70.0% isDDH (9, 10) or a ≥96% ANI (9) value was used as the cutoff to define a bacterial species.

### Phenotypic characterization for strains of two novel species.

Motility was examined using a CX21FS1 light microscope (Olympus, Tokyo, Japan). The Gram-staining reaction was performed as described previously (38). Growth at different temperatures (4, 15, 20, 25, 30, 35, 37, 45, and 50°C), at different pH values (3.0 to 12.0, at intervals of 1.0 pH unit), and at various salt concentrations (0 to 10% [wt/vol] NaCl) was determined in 15-ml test tubes containing 3 ml tryptic soy broth (TSB; Hopebio, Qingdao, China) after incubation for 2 days in a thermostatically controlled water bath as described previously (39). Anaerobic growth was performed by incubating cultures on nutrient agar for 7 days in an anaerobic bag (bioMérieux). Biochemical characteristics of the three strains were determined using the API 20E kit and API 50CH kit according to the manufacturer’s instructions (bioMérieux). Catalase activity was examined by bubble formation after dropping 3% (vol/vol) H_2_O_2_ on fresh biomass grown for 24 h on nutrient agar. Oxidase activity was determined using oxidase reagent (bioMérieux). All tests were carried out by incubating at 35°C unless indicated otherwise.

### Analysis of whole-cell fatty acids for strains of two novel species.

Whole-cell fatty acids of strains WCHECL1060^T^, 090040, and 090044^T^ were analyzed by Guangdong Institute of Microbiology (Guangzhou, Guangdong, China) as described previously (40).

### Antimicrobial susceptibility and antimicrobial resistance genes of strains of two novel species.

*In vitro* antimicrobial susceptibility tests were performed by Vitek II using broth microdilution. In addition, MICs of colistin, imipenem, and meropenem were also determined using the microdilution broth method of the Clinical and Laboratory Standards Institute (CLSI) (41). Breakpoints defined by CLSI (41) were applied except for tigecycline, for which breakpoints defined by the European Committee on Antimicrobial Susceptibility Testing (EUCAST; http://www.eucast.org/) were used. Antimicrobial resistance genes of clinical strains WCHECL1060^T^, 090040, and 090044^T^ were identified from genome sequences using the ABRicate program v1.0.1 (https://github.com/tseemann/abricate) to query the ResFinder database (http://genomicepidemiology.org/, accessed 16 April 2020).

### Curation of species identification for *Enterobacter* genome species in GenBank.

We used txid547 [Organism:exp] AND “latest refseq” [filter] to search NCBI GenBank and found 1,997 genome sequences labeled *Enterobacter* (Data Set S2 in the supplemental material, accessed 16 April 2020). All of the 1,997 sequences were retrieved and were then subjected to precise species identification using ANI and isDDH as described above. Strains that have a <70% isDDH value and a <96% ANI value with any known *Enterobacter* species are likely to belong to a novel species, which is temporarily assigned a taxon here as the establishment of a novel species requires phenotypic characterizations in addition to genome analysis.

### Data availability.

The draft whole-genome sequences of strains ATCC 23373^T^, WCHECL1060^T^, 090040, and 090044^T^ have been deposited into DDBJ/EMBL/GenBank under accession numbers WJWQ00000000, LFDQ00000000, RXSJ00000000, and RXRX00000000, respectively. Whole-genome sequences of the type strains of all species and subspecies within the genus *Enterobacter* and all other species of the family *Enterobacteriaceae* retrieved from the NCBI database are listed in Data Set S1. The 1,997 genome sequences labeled as *Enterobacter* in GenBank (accessed 16 April 2020) are listed in Data Set S2.
