# Supplementary material for: Precise Species Identification for Enterobacter: a Genome Sequence-Based Study with Reporting of Two Novel Species, Enterobacter quasiroggenkampii sp. nov. and Enterobacter quasimori sp. nov
Source: mSystems. 2020 Aug 4;5(4):e00527-20. doi: 10.1128/mSystems.00527-20 (PMC7406230; doi:10.1128/mSystems.00527-20)
Supplement: TABLE S1 [file mSystems.00527-20-st001.docx]

**Table S1.** Average nucleotide identity (ANI)/*in silico* DNA-DNA hybridization (isDDH) values between type strains of species belonging to the genus *Enterobacte*r, *Lelliottia* and *Leclercia*.

|  | 1 | 2 | 3 | 4 | 5 | 6 | 7 | 8 | 9 | 10 | 11 | 12 | 13 | 14 | 15 | 16 | 17 | 18 | 19 | 20 | 21 | 22 | 23 | 24 | 25 | 26 | 27 |
| --- | --- | --- | --- | --- | --- | --- | --- | --- | --- | --- | --- | --- | --- | --- | --- | --- | --- | --- | --- | --- | --- | --- | --- | --- | --- | --- | --- |
| 1 | - | 87.64/34.5 | 87.58/34.8 | 89.57/40.2 | 88.98/38.1 | 87.59/34.9 | 89.91/40.9 | 89.02/38.0 | 86.35/32.3 | 89.12/38.7 | 89.06/38.5 | 87.54/34.4 | 87.65/35.0 | 88.85/37.6 | 88.03/36.1 | 87.00/33.0 | 95.55/66.8 | 89.55/39.8 | 88.32/36.4 | 85.85/31.0 | 82.04/25.8 | 87.68/35.0 | 87.58/34.9 | 82.38/26.0 | 82.24/25.7 | 82.98/26.8 | 83.22/27.1 |
| 2 | 87.52/34.5 | - | 86.45/32.1 | 87.92/35.9 | 86.15/33.4 | 86.15/32.1 | 88.39/36.7 | 87.81/35.4 | 85.41/30.7 | 87.66/35.4 | 87.73/35.6 | 94.92/62.0 | 86.27/32.4 | 86.39/32.8 | 87.38/34.9 | 87.36/34.4 | 87.45/34.0 | 88.01/36.1 | 88.80/37.1 | 85.37/30.4 | 81.58/25.50 | 86.35/32.5 | 86.55/33.2 | 81.79/25.7 | 81.77/25.3 | 82.59/26.4 | 82.77/26.4 |
| 3 | 87.76/34.8 | 86.15/32.1 | - | 86.91/33.5 | 87.13/34.0 | 93.41/53.6 | 87.22/34.4 | 87.88/35.3 | 86.04/31.5 | 86.90/33.7 | 87.56/35.0 | 86.40/32.5 | 94.13/58.0 | 87.20/33.5 | 86.72/33.4 | 85.98/31.4 | 87.22/34.1 | 87.17/34.2 | 86.58/32.6 | 85.26/30.0 | 82.12/25.9 | 95.71/66.6 | 86.93/33.8 | 82.20/25.9 | 82.14/25.4 | 82.92/26.7 | 83.12/26.6 |
| 4 | 89.68/40.2 | 88.16/35.9 | 86.84/33.5 | - | 87.82/35.7 | 86.93/33.6 | 92.88/51.7 | 90.76/43.1 | 85.76/31.5 | 92.19/49.3 | 90.49/42.7 | 87.72/35.8 | 86.86/33.7 | 87.36/34.5 | 89.66/40.6 | 87.73/34.9 | 88.49/37.3 | 95.37/64.8 | 91.10/44.7 | 85.51/30.8 | 82.03/25.9 | 87.01/34.0 | 86.80/33.9 | 82.00/26.0 | 82.01/25.5 | 82.87/26.8 | 83.11/27.0 |
| 5 | 88.91/38.1 | 86.88/33.4 | 86.97/34.0 | 87.62/35.7 | - | 87.08/33.9 | 88.67/37.7 | 88.09/35.8 | 86.08/32.0 | 87.60/34.8 | 88.10/36.2 | 86.62/33.2 | 87.05/33.9 | 89.54/39.7 | 86.51/32.7 | 86.56/32.7 | 88.47/37.9 | 87.89/36.0 | 87.06/34.1 | 85.55/30.7 | 82.43/25.8 | 87.21/33.8 | 88.32/34.0 | 82.23/26.0 | 82.35/25.7 | 83.22/26.8 | 83.28/27.1 |
| 6 | 87.63/34.9 | 86.15/32.1 | 93.50/53.6 | 86.97/33.6 | 87.25/33.9 | - | 87.31/34.2 | 88.21/36.7 | 85.76/31.7 | 86.72/32.0 | 87.86/35.3 | 86.09/32.1 | 92.79/51.6 | 86.80/33.5 | 86.30/32.2 | 85.76/31.3 | 86.94/34.1 | 87.23/34.3 | 86.15/32.4 | 85.03/29.8 | 82.41/26.0 | 93.10/52.8 | 87.98/33.5 | 82.12/26.0 | 82.13/25.5 | 82.91/26.7 | 83.12/26.8 |
| 7 | 90.07/40.9 | 88.38/36.7 | 87.34/34.4 | 93.25/51.7 | 88.79/37.7 | 87.35/34.2 | - | 91.61/44.9 | 86.94/32.3 | 93.34/52.4 | 91.74/45.6 | 88.92/37.2 | 87.95/34.6 | 88.59/36.1 | 90.85/42.7 | 88.61/36.1 | 90.08/39.9 | 93.02/51.7 | 91.67/45.2 | 86.35/31.2 | 83.29/26.3 | 87.99/34.8 | 88.37/34.8 | 83.33/26.5 | 82.99/25.8 | 83.96/27.5 | 83.58/27.3 |
| 8 | 88.95/38.0 | 87.81/35.4 | 87.59/35.3 | 91.02/43.1 | 88.01/35.8 | 88.20/36.7 | 91.50/44.9 | - | 86.52/31.7 | 90.63/42.4 | 93.64/53.8 | 88.24/35.6 | 88.17/35.5 | 87.87/34.7 | 90.90/43.0 | 87.85/34.5 | 88.76/36.9 | 90.81/43.9 | 89.86/39.8 | 85.82/30.4 | 82.92/25.9 | 88.24/35.5 | 88.09/33.9 | 82.99/25.9 | 82.69/25.5 | 83.53/26.9 | 83.16/26.9 |
| 9 | 86.33/32.3 | 85.41/30.7 | 85.74/31.5 | 86.39/31.5 | 86.07/32.0 | 85.74/31.7 | 86.84/32.3 | 86.55/31.7 | - | 86.44/31.8 | 86.57/31.7 | 85.97/30.8 | 86.37/31.5 | 86.65/32.0 | 86.21/31.2 | 85.69/30.4 | 86.51/31.9 | 85.89/32.0 | 86.21/31.2 | 85.37/29.4 | 82.62/25.4 | 86.37/31.5 | 87.42/32.1 | 82.81/25.8 | 82.61/25.3 | 83.25/26.4 | 82.92/26.6 |
| 10 | 89.10/38.7 | 87.66/35.4 | 86.74/33.7 | 92.19/49.3 | 87.63/34.8 | 86.70/32.0 | 93.28/52.4 | 90.66/42.4 | 86.50/31.8 | - | 90.76/42.5 | 88.27/35.8 | 87.59/34.0 | 87.93/34.8 | 90.89/42.9 | 87.89/34.6 | 88.92/37.3 | 91.80/48.6 | 90.75/42.4 | 85.88/30.6 | 82.91/26.0 | 87.43/33.8 | 87.98/36.7 | 83.07/26.4 | 82.65/25.5 | 83.56/27.0 | 83.08/27.1 |
| 11 | 89.25/38.5 | 87.73/35.6 | 87.58/35.0 | 90.49/42.7 | 88.12/36.2 | 87.85/35.3 | 91.69/45.6 | 93.69/53.8 | 86.59/31.7 | 90.79/42.5 | - | 88.36/35.6 | 88.21/35.4 | 88.14/35.1 | 90.72/42.4 | 87.92/34.7 | 89.17/37.7 | 90.78/43.3 | 89.95/39.8 | 85.99/30.6 | 82.96/25.9 | 88.14/35.2 | 88.24/34.1 | 83.06/26.0 | 82.76/25.6 | 83.63/26.9 | 83.31/27.0 |
| 12 | 87.30/34.4 | 94.92/62.0 | 86.06/32.5 | 88.28/35.8 | 86.66/33.2 | 86.13/32.1 | 88.79/37.2 | 88.27/35.6 | 86.01/30.8 | 88.30/35.8 | 88.33/35.6 | - | 86.97/32.9 | 86.91/32.4 | 88.21/35.7 | 87.66/34.2 | 87.64/34.0 | 87.57/36.2 | 88.90/36.9 | 85.83/30.3 | 82.64/25.6 | 86.86/32.6 | 87.88/33.2 | 82.90/26.1 | 82.59/25.4 | 83.40/26.7 | 82.69/26.6 |
| 13 | 87.77/35.0 | 86..27/32.4 | 94.09/58.0 | 87.51/33.7 | 87.12/33.9 | 92.85/51.6 | 87.88/34.6 | 88.33/35.5 | 86.41/31.5 | 87.72/34.0 | 88.25/35.4 | 87.06/32.9 | - | 87.59/33.7 | 87.42/33.7 | 86.36/31.6 | 87.73/34.2 | 87.15/34.3 | 87.05/32.6 | 85.81/30.2 | 82.97/26.1 | 94.79/60.0 | 88.15/33.8 | 83.00/26.0 | 82.75/25.5 | 83.53/26.8 | 83.14/26.7 |
| 14 | 88.72/37.6 | 86.39/32.8 | 86.97/33.5 | 87.36/34.5 | 89.58/39.7 | 86.88/33.5 | 88.50/36.1 | 87.87/34.7 | 86.71/32.0 | 87.90/34.8 | 88.04/35.1 | 86.88/32.4 | 87.54/33.7 | - | 87.22/33.2 | 86.62/31.8 | 88.95/37.2 | 87.18/34.8 | 87.37/33.5 | 86.15/30.8 | 82.76/25.6 | 87.53/33.6 | 88.45/34.2 | 82.93/25.8 | 82.82/25.6 | 83.50/26.7 | 83.00/26.8 |
| 15 | 88.19/36.1 | 87.38/34.9 | 86.73/33.4 | 90.26/40.6 | 86.55/32.7 | 86.32/32.2 | 90.83/42.7 | 91.03/43.0 | 86.29/31.2 | 90.97/42.9 | 90.78/42.4 | 88.21/35.7 | 87.47/33.7 | 87.26/33.2 | - | 87.77/34.6 | 88.27/35.4 | 89.87/41.3 | 89.72/39.2 | 85.74/30.1 | 82.68/25.5 | 87.38/33.4 | 87.78/33.3 | 82.91/26.1 | 82.63/25.5 | 83.52/26.9 | 82.90/26.6 |
| 16 | 86.91/33.0 | 87.36/34.4 | 85.76/31.4 | 88.03/34.9 | 86.59/32.7 | 85.81/31.3 | 88.53/36.1 | 87.92/34.5 | 85.80/30.4 | 87.90/34.6 | 87.94/34.7 | 87.72/34.2 | 86.34/31.6 | 86.62/31.8 | 87.75/34.6 | - | 87.04/32.6 | 87.81/35.5 | 88.03/34.6 | 85.79/30.3 | 82.30/25.1 | 86.41/31.5 | 87.69/32.7 | 82.58/25.4 | 82.63/25.3 | 83.29/26.6 | 82.77/26.4 |
| 17 | 95.32/66.8 | 87.45/34.0 | 86.86/34.1 | 88.98/37.3 | 88.46/37.9 | 86.91/34.1 | 89.88/39.9 | 88.77/36.9 | 86.50/31.9 | 88.87/37.3 | 89.04/37.7 | 87.58/34.0 | 87.59/34.2 | 88.89/37.2 | 88.14/35.4 | 86.92/32.6 | - | 88.26/37.5 | 88.20/35.2 | 86.04/30.8 | 82.50/25.5 | 87.67/34.2 | 88.61/34.7 | 82.67/25.7 | 82.69/25.5 | 83.45/26.8 | 82.82/26.8 |
| 18 | 89.62/39.8 | 88.01/36.1 | 87.17/34.2 | 95.37/64.8 | 87.99/36.0 | 87.26/34.3 | 92.85/51.7 | 91.08/43.9 | 86.07/32.0 | 92.04/48.6 | 90.67/43.3 | 87.96/36.2 | 87.15/34.3 | 87.42/34.8 | 89.91/41.3 | 87.83/35.5 | 88.53/37.5 | - | 91.35/45.5 | 85.47/30.8 | 82.23/26.1 | 87.42/34.7 | 88.25/34.4 | 82.26/26.2 | 82.10/25.6 | 83.21/27.1 | 83.21/27.1 |
| 19 | 88.07/36.4 | 88.80/37.1 | 86.58/32.6 | 91.10/44.7 | 87.02/34.1 | 86.17/32.4 | 91.49/45.2 | 89.85/39.8 | 86.12/31.2 | 90.64/42.4 | 89.86/39.8 | 88.85/36.9 | 86.92/32.6 | 87.31/33.5 | 89.58/39.2 | 87.96/34.6 | 88.20/35.2 | 91.06/45.5 | - | 85.74/30.3 | 82.57/25.5 | 86.97/32.8 | 87.64/36.0 | 82.77/25.8 | 82.52/25.4 | 83.32/26.7 | 82.98/26.8 |
| 20 | 85.70/31.0 | 85.37/30.4 | 85.26/30.0 | 85.93/30.8 | 85.58/30.7 | 85.00/29.8 | 86.25/31.2 | 85.86/30.4 | 85.41/29.4 | 85.87/30.6 | 85.96/30.6 | 85.79/30.3 | 85.70/30.2 | 86.14/30.8 | 85.68/30.1 | 85.73/30.3 | 86.03/30.8 | 85.22/30.8 | 85.79/30.3 | - | 81.98/24.7 | 85.72/30.2 | 87.12/31.6 | 82.47/25.3 | 82.78/25.5 | 83.47/26.7 | 82.86/26.5 |
| 21 | 82.41/25.8 | 81.58/25.5 | 82.12/25.9 | 82.03/25.9 | 82.42/25.8 | 82.45/26.0 | 83.02/26.3 | 82.97/25.9 | 82.67/25.4 | 82.92/26.0 | 82.94/25.9 | 82.59/25.6 | 82.89/26.1 | 82.76/25.6 | 82.54/25.5 | 82.21/25.1 | 82.54/25.5 | 82.42/26.1 | 82.67/25.5 | 82.04/24.7 | - | 82.92/26.0 | 83.78/25.5 | 81.97/24.7 | 81.41/23.8 | 82.21/25.0 | 82.11/25.3 |
| 22 | 87.74/35.0 | 86.35/32.5 | 95.71/66.6 | 87.54/34.0 | 87.22/33.8 | 93.12/52.8 | 87.89/34.8 | 88.32/35.5 | 86.42/31.5 | 87.46/33.8 | 88.15/35.2 | 86.92/32.6 | 94.77/60.0 | 87.56/33.6 | 87.35/33.4 | 86.39/31.5 | 87.81/34.2 | 87.33/34.7 | 87.05/32.8 | 85.80/30.2 | 82.94/26.0 | - | 88.10/33.8 | 82.91/25.9 | 82.72/25.5 | 83.50/26.7 | 83.16/26.6 |
| 23 | 87.81/34.9 | 86.55/33.2 | 86.93/33.8 | 87.14/33.9 | 88.32/34.0 | 87.98/33.5 | 88.37/34.8 | 88.09/33.9 | 87.42/32.1 | 87.98/36.7 | 88.24/34.1 | 87.88/33.2 | 88.15/33.8 | 88.45/34.2 | 87.78/33.3 | 87.69/32.7 | 88.61/34.7 | 88.25/34.4 | 87.64/36.0 | 87.12/31.6 | 83.78/25.5 | 88.10/33.8 | - | 84.10/25.9 | 83.92/25.8 | 84.89/27.0 | 83.45/27.1 |
| 24 | 82.35/26.0 | 81.79/25.7 | 82.20/25.9 | 81.94/26.0 | 82.22/26.0 | 82.15/26.0 | 83.10/26.5 | 82.97/25.9 | 82.80/25.8 | 83.06/26.4 | 83.03/26.0 | 82.80/26.1 | 82.84/26.0 | 82.92/25.8 | 82.74/26.1 | 82.39/25.4 | 82.65/25.7 | 82.08/26.2 | 82.73/25.8 | 82.48/25.3 | 81.91/24.7 | 82.80/25.9 | 84.10/25.9 | - | 81.74/24.4 | 82.59/25.5 | 81.96/25.6 |
| 25 | 82.33/25.7 | 81.77/25.3 | 82.14/25.4 | 81.98/25.5 | 82.37/25.7 | 82.14/25.5 | 82.83/25.8 | 82.69/25.5 | 82.57/25.3 | 82.64/26.4 | 82.69/25.6 | 82.55/25.4 | 82.62/25.5 | 82.82/25.6 | 82.50/25.5 | 82.49/25.3 | 82.71/25.5 | 82.16/25.6 | 82.56/25.4 | 82.78/25.5 | 81.37/23.8 | 82.63/25.5 | 83.92/25.8 | 81.77/24.4 | - | 84.96/29.0 | 84.59/28.8 |
| 26 | 83.09/26.8 | 82.81/26.4 | 82.79/26.7 | 82.79/26.8 | 83.20/26.8 | 82.90/26.7 | 83.85/27.5 | 83.58/26.9 | 83.43/26.4 | 83.61/27.0 | 83.68/26.9 | 83.43/26.7 | 83.54/26.8 | 83.62/26.7 | 83.47/26.9 | 83.25/26.6 | 83.59/26.8 | 83.17/27.1 | 83.46/26.7 | 83.59/26.7 | 82.28/25.0 | 83.47/26.7 | 84.89/27.0 | 82.71/25.5 | 85.07/29.0 | - | 88.92/38.2 |
| 27 | 83.25/27.1 | 82.96/26.4 | 82.92/26.6 | 83.02/27.0 | 83.26/27.1 | 83.08/26.8 | 83.4/27.3 | 83.21/26.9 | 83.02/26.6 | 83.19/27.1 | 83.16/27.0 | 82.8/26.6 | 83.05/26.7 | 83.15/26.8 | 82.79/26.6 | 82.74/26.4 | 82.99/26.8 | 83.19/27.1 | 83.15/26.8 | 83.02/26.5 | 81.97/25.3 | 83.01/26.6 | 83.30/27.1 | 82.02/25.6 | 84.50/28.8 | 88.97/38.0 | - |

Species and type strains: 1, *E. quasimori* 090044^T^; 2, *E. dissolvens* ATCC 23373^T^; 3, *E. hoffmannii* DSM 14563^T^; 4, *E. quasiroggenkampi* WCHECL1060^T^; 5, *E. wuhouensis* WCHEs120002^T^; 6, *E. quasihormaechei* WCHEs120003^T^; 7, *E. asburiae* JCM 6051^T^; 8, *E. bugandensis* EB-247^T^; 9, *E. cancerogenus* LMG 2693^T^; 10, *E. chengduensis* WCHECL-C4^T^; 11, *E. chuandaensis* 090028^T^; 12, *E. cloacae* ATCC 13047^T^; 13, *E. hormaechei* NBRC 105718^T^; 14, *E. huaxiensis* 090008^T^; 15, *E. kobei* DSM 13645^T^; 16, *E. ludwigii* EN-119^T^; 17, *E. mori* LMG 25706^T^; 18, *E. roggenkampii* DSM 16690^T^; 19, *E. sichuanensis* WCHECL1597^T^; 20, *E. soli* ATCC BAA-2102^T^; 21, *Pseudenterobacter timonensis* mt20^T^; 22, *E. xiangfangensis* 10-17^T^; 23, *E. oligotrophica* CCA6^T^; 24, *Leclercia adecarboxylata* ATCC 23216^T^; 25, *Lelliottia amnigena* NBRC 105700^T^; 26, *Lelliottia jeotgali* PFL01^T^; 27, *Lelliottia nimipressuralis* CICC 24156^T^.
