# Supplementary figures and images for: Precise Species Identification for Enterobacter: a Genome Sequence-Based Study with Reporting of Two Novel Species, Enterobacter quasiroggenkampii sp. nov. and Enterobacter quasimori sp. nov
Source: mSystems. 2020 Aug 4;5(4):e00527-20. doi: 10.1128/mSystems.00527-20 (PMC7406230; doi:10.1128/mSystems.00527-20)

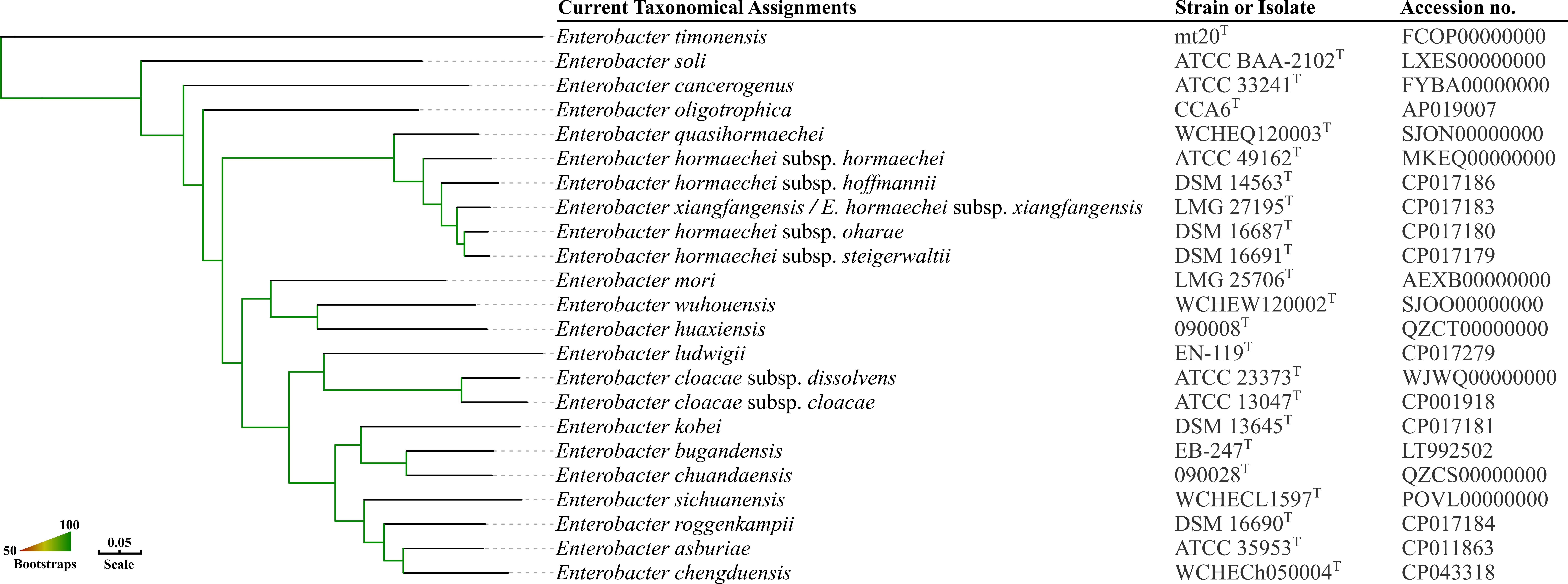

Supplement: FIG S1 [file mSystems.00527-20-sf001.tif]

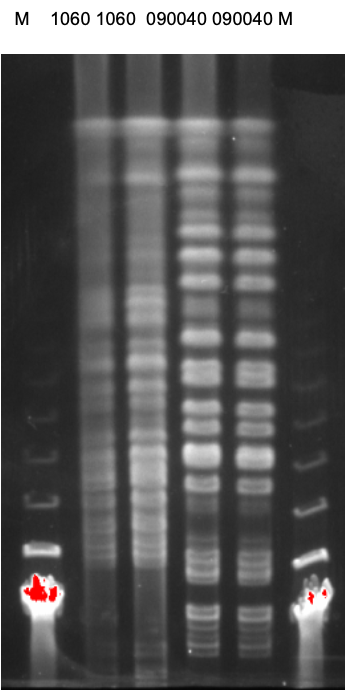

Supplement: FIG S2 [file mSystems.00527-20-sf002.tif]
