# Supplementary material for: Precise Species Identification for Enterobacter: a Genome Sequence-Based Study with Reporting of Two Novel Species, Enterobacter quasiroggenkampii sp. nov. and Enterobacter quasimori sp. nov
Source: mSystems. 2020 Aug 4;5(4):e00527-20. doi: 10.1128/mSystems.00527-20 (PMC7406230; doi:10.1128/mSystems.00527-20)
Supplement: TEXT S1 [file mSystems.00527-20-s0001.docx]

**Text S1 Antimicrobial susceptibility and resistance genes**

**Antimicrobial susceptibility and antimicrobial resistance genes of *E. quasiroggenkampi* strains WCHECL1060^T^ and 090040**

Both of the two strains WCHECL1060^T^ and 090040 were resistant to ampicillin, amoxicillin/clavulanic acid, cefazolin, cefoxitin, and colistin, and were susceptible to amikacin, aztreonam, cefepime, ceftriaxone, piperacillin/tazobactam, ciprofloxacin, gentamicin, trimethoprim/sulfamethoxazole, levofloxacin, tigecycline, and tobramycin (Table S3). Both strains had a *bla*_MIR_ gene encoding AmpC-type β-lactamases, which could explain their resistance to amoxicillin/clavulanic acid, cefazolin, and cefoxitin (1), *oqxA*/*oqxB* genes, which mediate resistance to chloramphenicol and olaquindox (a quinoxaline) (2), and fosfomycin-resistant gene *fosA*. Of note, both strains were high-level resistant to colistin (MIC, 256 or 512 mg/L). Our previous work has identified a novel gene, *ecr*, able to mediate high-level resistance to colistin (3), from WCHECL1060^T^ and this gene is also present in strain 090040. Strain 090040 was susceptible to imipenem and meropenem (Table S3). However, strain WCHECL1060^T^ was resistant to meropenem and imipenem although it was susceptible to cefepime and ceftriaxone. This peculiar susceptibility phenotype of strain WCHECL1060^T^ is due to the presence of *bla*_IMI-1_, which encodes IMI-1, a carbapenemase with strong activity against carbapenems but with only weak activity against third- or fourth-generation cephalosporins (4).

**Antimicrobial susceptibility and antimicrobial resistance genes of *E. quasimori* strain 090044^T^**

Strain 090044^T^ was resistant to ampicillin, amoxicillin/clavulanic acid, cefazolin, cefoxitin, and was susceptible to amikacin, aztreonam, cefepime, ceftriaxone, colistin, piperacillin/tazobactam, ciprofloxacin, gentamicin, trimethoprim/sulfamethoxazole, levofloxacin, tigecycline, and tobramycin (Table S3). Strain 090044^T^ carried a *bla*_ACT_ gene encoding AmpC-type β-lactamases, which could explain their resistance to amoxicillin/clavulanic acid, cefazolin, and cefoxitin (5). The strain also had *oqxA*/*oqxB* genes, fosfomycin-resistant gene *fosA*, and *qnrE1*, which leads to reduced susceptibility to quinolones (6).

**References**

1. **Jacoby GA, Tran J.** 1999. Sequence of the MIR-1 β-lactamase gene. Antimicrob Agents Chemother **43:**1759-1760.

2. **Hansen LH, Johannesen E, Burmolle M, Sorensen AH, Sorensen SJ.** 2004. Plasmid-encoded multidrug efflux pump conferring resistance to olaquindox in *Escherichia coli*. Antimicrob Agents Chemother **48:**3332-3337.

3. **Huang L, Feng Y, Zong Z.** 2019. Heterogeneous resistance to colistin in *Enterobacter cloacae* complex due to a new small transmembrane protein. J Antimicrob Chemother **74:**2551-2558. .

4. **Rasmussen BA, Bush K, Keeney D, Yang Y, Hare R, O'Gara C, Medeiros AA.** 1996. Characterization of IMI-1 β-lactamase, a class A carbapenem-hydrolyzing enzyme from *Enterobacter cloacae*. Antimicrob Agents Chemother **40:**2080-2086.

5. **Rottman M, Benzerara Y, Hanau-Bercot B, Bizet C, Philippon A, Arlet G.** 2002. Chromosomal *ampC* genes in *Enterobacter* species other than *Enterobacter cloacae*, and ancestral association of the ACT-1 plasmid-encoded cephalosporinase to *Enterobacter asburiae*. FEMS Microbiol Lett **210:**87-92.

6. **Albornoz E, Tijet N, De Belder D, Gomez S, Martino F, Corso A, Melano RG, Petroni A.** 2017. *qnrE1*, a member of a new family of plasmid-located quinolone resistance genes, originated from the chromosome of *Enterobacter* species. Antimicrob Agents Chemother **61**.
