# Supplementary material for: Precise Species Identification for Enterobacter: a Genome Sequence-Based Study with Reporting of Two Novel Species, Enterobacter quasiroggenkampii sp. nov. and Enterobacter quasimori sp. nov
Source: mSystems. 2020 Aug 4;5(4):e00527-20. doi: 10.1128/mSystems.00527-20 (PMC7406230; doi:10.1128/mSystems.00527-20)
Supplement: TABLE S2 [file mSystems.00527-20-st002.docx]

**Table S2.** Fatty acid profiles of the strain *E. qausiroggenkampi* WCHECL1060^T^, *E. qausiroggenkampi* 090040, *E. qausimori* 090044^T^ and other type strains of *Enterobacter* species.

| Fatty acid | 1 | 2 | 3 | 4 | 5 | 6 | 7 | 8 | 9 | 10 | 11 | 12 | 13 | 14 | 15 | 16 | 17 | 18 | 19 | 20 |
| --- | --- | --- | --- | --- | --- | --- | --- | --- | --- | --- | --- | --- | --- | --- | --- | --- | --- | --- | --- | --- |
| C_12:0_ | 2.0 | 1.5 | 4.3 | 4.5 | 3.5 | 3.8 | 3.9 | 3.9 | 2.8 | 1.0 | 3.9 | 2.5 | 4.1 | 3.2 | 4.0 | 3.9 | 3.0 | 2.5 | 2.3 | 3.7 |
| C_13:0_ | 0.8 | - | 0.4 | 0.7 | 0.4 | 0.3 | 0.6 | 0.4 | 0.3 | 0.7 | 1.1 | 0.6 | 0.5 | 0.1 | 0.9 | 0.6 | 0.7 | 1.2 | 0.4 | 1.4 |
| C_14:0_ | 10.9 | 9.6 | 6.4 | 8.8 | 6.3 | 5.8 | 6.2 | 7.7 | 12.4 | 8.3 | 7.6 | 6.7 | 7.3 | 7.9 | 6.2 | 6.5 | 6.5 | 8.4 | 9.7 | 5.6 |
| C_15:0_ | - | 3.3 | 2.4 | - | 2.3 | 2.0 | - | - | - | - | - | - | - | 1.1 | - | - | - | - | - | - |
| C_16:0_ | 25.2 | 30.9 | 30.21 | 23.2 | 25.0 | 27.8 | 27.2 | 29.4 | 27.1 | 27.4 | 27.3 | 29.3 | 29.6 | 30.8 | 25.7 | 30.3 | 27.9 | 21.8 | 30.2 | 22.7 |
| C_17:0_ | 2.5 | 1.9 | 2.0 | 4.2 | 1.8 | 1.6 | 2.6 | 1.7 | 1.2 | 4.1 | 2.4 | 3.4 | 2.5 | 1.0 | 3.2 | 4.1 | 3.4 | 4.9 | 2.0 | 4.0 |
| C_17:0_ cyclo | 22.3 | 11.4 | 15.9 | 6.7 | 17.3 | 16.2 | 21.0 | 11.9 | 19.4 | 17.9 | 12.4 | 20.4 | 22.9 | 9.6 | 25.2 | 25.6 | 26.0 | 21.1 | 21.7 | 14 |
| C_18:0_ | 0.2 | 0.9 | 0.8 | - | 0.6 | 0.6 | 0.6 | 0.7 | 0.2 | 0.3 | - | 0.5 | 0.7 | 0.5 | 0.4 | 0.7 | 0.5 | 0.4 | 0.5 | 0.3 |
| C_18:1ω7c_ | 12.9 | 14.3 | 17.0 | 18.3 | 25.5 | 21.9 | 18.1 | 21.1 | 12.2 | 17.6 | 16.1 | 14.3 | 16.3 | 22.0 | 15.7 | 12.9 | 13.4 | 20.6 | 15.9 | 21.3 |
| C_19:0_ cyclo _ω8c_ | 2.0 | 0.6 | 0.7 | - | 2.0 | 0.7 | 2.5 | 1.5 | 1.4 | 0.8 | - | 6.1 | 3.6 | 0.6 | 6.0 | 5.1 | 7.0 | 4.4 | 5.8 | 3.3 |
| Sum of iso-C_15:1_ H/C_13:0_ 3-OH | 1.1 | - | - | 3.0 | 0.1 | 0.1 | - | - | 0.4 | 1.0 | 1.7 | 0.6 | 0.4 | - | 0.7 | 0.6 | 0.9 | 1.5 | 0.3 | 1.3 |
| Sum of iso-C_16:1_ I/C_14:0_ 3-OH | 8.2 | 11.5 | 9.3 | 13.0 | - | - | 7.9 | 9.3 | 12.3 | 7.9 | 8.9 | 6.9 | 6.9 | 7.7 | 6.8 | 6.5 | 6.1 | 6.2 | 6.8 | 8.1 |
| Sum of C_16:1ω7c_/C_16:1ω6c_ | 9.0 | 9.6 | 9.6 | 23.0 | 6.8 | 11.1 | 6.7 | 9.9 | 9.0 | 9.9 | 16.3 | 6.5 | 5.0 | 14.6 | 4.9 | 2.9 | 3.5 | 5.7 | 4.5 | 12.6 |

Species and type strains: 1, *E. qausiroggenkampi* WCHECL1060^T^; 2, *E. qausiroggenkampi* 090040; 3, *E. qausimori* 090044^T^; 4, *E. dissolvens* ATCC 23373^T^; 5, *E. wuhouensis* WCHEs120002^T^; 6, *E. quasihormaechei* WCHEs120003^T^; 7, *E. huaxiensis* 090008^T^; 8, *E. chuandaensis* 090028^T^; 9, *E. sichuanensis* WCHECL1597^T^; 10, *E. chengduensis* WCHECL-C4^T^; 11, *E. soli* ATCC BAA-2102^T^; 12, *E. cloacae* ATCC 13047^T^; 13, *E. mori* LMG 25706^T^; 14, *E. bugandensis* EB-247^T^; 15, *E. ludwigii* EN-119^T^; 16, *E. cancerogenus* LMG 2693^T^; 17, *E. asburiae* JCM 6051^T^; 18, *E. hormaechei* NBRC 105718^T^; 19, *E. xiangfangensis* 10-17^T^; 20, *E. oligotrophica* CCA6^T^.

-, Not detected.

Data for type strains of each species other than *E. qausiroggenkampi* WCHECL1060^T^, *E. qausiroggenkampi* 090040 and *E. qausimori* 090044^T^ are from references (1-7).

**References**

1. **Manter DK, Hunter WJ, Vivanco JM.** 2011. *Enterobacter soli* sp. nov.: a lignin-degrading gamma-proteobacteria isolated from soil. Curr Microbiol **62:**1044-1049.

2. **Doijad S, Imirzalioglu C, Yao Y, Pati NB, Falgenhauer L, Hain T, Foesel BU, Abt B, Overmann J, Mirambo MM, Mshana SE, Chakraborty T.** 2016. *Enterobacter bugandensis* sp. nov., from neonatal blood. Int J Syst Evol Microbiol **66:**968-974.

3. **Wu W, Yu Y, Zong Z.** 2018. *Enterobacter sichuanensis* sp. nov., recovered from human urine. Int J Syst Evol Microbiol **68:**3922-3927.

4. **Wu W, Feng Y, Zong Z.** 2019. Characterization of a strain representing a new *Enterobacter* species, *Enterobacter chengduensis* sp. nov. Antonie Van Leeuwenhoek**:**491-500.

5. **Wang C, Wu W, Wei L, Feng Y, Kang M, Xie Y, Zong Z.** 2019. Enterobacter wuhouensis sp. nov. and Enterobacter quasihormaechei sp. nov. recovered from human sputum. Int J Syst Evol Microbiol.

6. **Akita H, Matsushika A, Kimura ZI.** 2019. Enterobacter oligotrophica sp. nov., a novel oligotroph isolated from leaf soil. Microbiologyopen **8:**e843.

7. **Kampfer P, McInroy JA, Glaeser SP.** 2015. *Enterobacter muelleri* sp. nov., isolated from the rhizosphere of Zea mays. Int J Syst Evol Microbiol **65:**4093-4099.
