# Supplementary material for: Precise Species Identification for Enterobacter: a Genome Sequence-Based Study with Reporting of Two Novel Species, Enterobacter quasiroggenkampii sp. nov. and Enterobacter quasimori sp. nov
Source: mSystems. 2020 Aug 4;5(4):e00527-20. doi: 10.1128/mSystems.00527-20 (PMC7406230; doi:10.1128/mSystems.00527-20)
Supplement: TABLE S3 [file mSystems.00527-20-st003.docx]

**Table S3.** Antimicrobial susceptibility of strains WCHECL1060^T^, 090040 and 090044^T^.

| Antimicrobial | WCHECL1060^T^ | | 090040 |  | 090044 |  |
| --- | --- | --- | --- | --- | --- | --- |
|  | MIC  (mg/L) | Category^$^ | MIC (mg/L) | Category^$^ | MIC (mg/L) | Category^$^ |
| Ampicillin | ≥32 | R | ≥32 | R | ≥32 | R |
| Amikacin | ≤2 | S | ≤2 | S | ≤2 | S |
| Amoxicillin/clavulanic acid | ≥32 | R | ≥32 | R | ≥32 | R |
| Aztreonam | ≤1 | S | ≤1 | S | ≤1 | S |
| Cefazolin | ≥64 | R | ≥64 | R | ≥64 | R |
| Cefepime | ≤1 | S | ≤1 | S | ≤1 | S |
| Cefoxitin | ≥64 | R | ≥64 | R | ≥64 | R |
| Ceftriaxone | ≤1 | S | ≤1 | S | ≤1 | S |
| Ciprofloxacin | ≤0.25 | S | ≤0.25 | S | ≤0.25 | S |
| Colistin | 256 | R | 512 | R | 1 | S |
| Gentamicin | ≤1 | S | ≤1 | S | ≤1 | S |
| Imipenem | 256 | R | ≤1 | S | ≤1 | S |
| Levofloxacin | ≤0.12 | S | ≤0.25 | S | ≤0.25 | S |
| Meropenem | 256 | R | ≤1 | S | ≤1 | S |
| Nitrofurantoin | 64 | I | 32 | S | 32 | S |
| Piperacillin/tazobactam | ≤4 | S | ≤4 | S | ≤4 | S |
| Tigecycline^#^ | ≤0.5 | S | ≤0.5 | S | ≤0.5 | S |
| Tobramycin | ≤1 | S | ≤1 | S | ≤1 | S |
| Trimethoprim/sulfamethoxazole | ≤1/19 | S | ≤1/19 | S | ≤1/19 | S |

^#^The susceptibility category of tigecycline was interpreted using the breakpoints of EUCAST.

^$^S, susceptible; I, intermediate; R, resistant.
