# Supplementary material for: Precise Species Identification for Enterobacter: a Genome Sequence-Based Study with Reporting of Two Novel Species, Enterobacter quasiroggenkampii sp. nov. and Enterobacter quasimori sp. nov
Source: mSystems. 2020 Aug 4;5(4):e00527-20. doi: 10.1128/mSystems.00527-20 (PMC7406230; doi:10.1128/mSystems.00527-20)
Supplement: TABLE S4 [file mSystems.00527-20-st004.docx]

**Table S4.** Tentative taxon assignations for new, unnamed non-*Enterobacter* species of the family *Enterobacteriaceae*

| Taxon | Accession no. | Strain | Closest species | ANI, % | DDH, % |
| --- | --- | --- | --- | --- | --- |
| A | CP000653 | 638 | *Lelliottia amnigena* | 93.643 | 54.10 |
| B | AKXM00000000 | Ag1 | *Cedecea neteri* | 91.909 | 45.10 |
| C | CP005991 | R4-368 | *Kosakonia sacchari* | 94.575 | 57.70 |
| D | AXOM00000000 | S611 | *Kosakonia cowanii* | 95.906 | 65.50 |
| E | JXAG00000000 | Bisph1 | *Kosakonia pseudosacchari* | 86.417 | 31.00 |
| F | JSWZ00000000 | CH1 | *Enterobacter cancerogenus* | 84.588 | 26.30 |
| G | CP019113 | SA187 | *Pseudescherichia vulneris* | 82.605 | 24.30 |
| H | NISF00000000 | 10-1 | *Raoultella terrigena* | 94.254 | 57.20 |
| I | PVZP00000000 | FS01 | *Leclercia adecarboxylata* | 91.745 | 44.80 |
| J | RCAA00000000 | R1(2018) | *Cedecea colo* | 87.527 | 33.50 |
| K | RJKQ00000000 | BIGb0383 | *Enterobacter quasimori* | 81.937 | 22.50 |
| L | SNZD00000000 | OV724 | *Lelliottia amnigena* | 93.361 | 52.50 |
| M | VCBB00000000 | MF024 | *Leclercia adecarboxylata* | 89.539 | 37.40 |
| N | VEJO00000000 | DE0047 | *Pseudescherichia vulneris* | 93.858 | 55.00 |
| O | VWWV00000000 | Cy-643 | *Cedecea neteri* | 91.647 | 44.20 |
| P | FOYH00000000 | kpr-6 | *Pseudescherichia vulneris* | 82.362 | 23.80 |
| Q | FTPH00000000 | Kh17 | *Pseudescherichia vulneris* | 82.373 | 23.80 |
| R | OBEF00000000 | CC120223-11 | *Scandinavium goeteborgense* | 85.361 | 28.20 |
